# Restoring Prostacyclin/PGI2-PTGIR signaling alleviates intestinal fibrosis in Crohn’s disease via fibroblast-specific YAP/TAZ inhibition

**DOI:** 10.1093/ecco-jcc/jjaf084

**Published:** 2025-05-20

**Authors:** Weijun Ou, Yaosheng Wang, Weimin Xu, Zhebin Hua, Xiaolei Wang, Wensong Ge, Wenjun Ding, Yingwei Chen, Chen-Ying Liu, Peng Du

**Affiliations:** Department of Colorectal Surgery, Xinhua Hospital, Shanghai Jiaotong University, School of Medicine, Shanghai, China; Shanghai Colorectal Cancer Research Center, Xinhua Hospital Affiliated to Shanghai Jiao Tong University School of Medicine, Shanghai, China; Department of Colorectal Surgery, Xinhua Hospital, Shanghai Jiaotong University, School of Medicine, Shanghai, China; Shanghai Colorectal Cancer Research Center, Xinhua Hospital Affiliated to Shanghai Jiao Tong University School of Medicine, Shanghai, China; Department of Colorectal Surgery, Xinhua Hospital, Shanghai Jiaotong University, School of Medicine, Shanghai, China; Shanghai Colorectal Cancer Research Center, Xinhua Hospital Affiliated to Shanghai Jiao Tong University School of Medicine, Shanghai, China; Department of Colorectal Surgery, Xinhua Hospital, Shanghai Jiaotong University, School of Medicine, Shanghai, China; Shanghai Colorectal Cancer Research Center, Xinhua Hospital Affiliated to Shanghai Jiao Tong University School of Medicine, Shanghai, China; Department of Gastroenterology, Shanghai Tenth People’s Hospital of Tongji University, Shanghai, China; Department of Gastroenterology, Xinhua Hospital, Shanghai Jiaotong University, School of Medicine, Shanghai, China; Department of Colorectal Surgery, Xinhua Hospital, Shanghai Jiaotong University, School of Medicine, Shanghai, China; Shanghai Colorectal Cancer Research Center, Xinhua Hospital Affiliated to Shanghai Jiao Tong University School of Medicine, Shanghai, China; Department of Gastroenterology, Xinhua Hospital, Shanghai Jiaotong University, School of Medicine, Shanghai, China; Department of Colorectal Surgery, Xinhua Hospital, Shanghai Jiaotong University, School of Medicine, Shanghai, China; Shanghai Colorectal Cancer Research Center, Xinhua Hospital Affiliated to Shanghai Jiao Tong University School of Medicine, Shanghai, China; Department of Colorectal Surgery, Xinhua Hospital, Shanghai Jiaotong University, School of Medicine, Shanghai, China; Shanghai Colorectal Cancer Research Center, Xinhua Hospital Affiliated to Shanghai Jiao Tong University School of Medicine, Shanghai, China

**Keywords:** Crohn’s disease, intestinal fibrosis, prostacyclin

## Abstract

**Background and Aims:**

Intestinal obstruction caused by fibrosis is a common and serious complication of Crohn’s disease (CD). Yes-associated protein (YAP) and transcriptional coactivator with PDZ-binding motifs (TAZ), the transcriptional effectors of the Hippo signaling pathway, have emerged as key drivers of intestinal fibrosis. Systematic inhibition of YAP/TAZ failed to combat fibrotic progression, probably due to the vital role of epithelial YAP/TAZ in intestinal homeostasis.

**Methods:**

Enzyme-Linked Immunosorbent Assay (ELISA) and immunohistochemical staining were used to detect serum Prostaglandin I2 (PGI2) levels and PGI2 Receptor (PTGIR) in clinical samples derived from CD patients. Dual luciferase reporter and Cut & Run assays were performed to explore the transcriptional regulatory mechanisms of PTGIR and PGI2 synthase (PTGIS) by tumor necrosis factor α (TNF-α) and transforming growth factor-beta (TGF-β), respectively. Primary intestinal fibroblasts and a chronic colitis model were used for assessing the efficacy of a PTGIR agonist in combating fibrosis.

**Results:**

The Gαs-coupled PTGIR is expressed in intestinal fibroblasts but is barely expressed in intestinal epithelial cells. PTGIR transcription is directly activated by p65 in fibroblasts upon TNF-α stimulation. Importantly, PTGIS is transcriptionally suppressed by TGF-β, leading to the loss of endogenous antifibrotic PGI2-PTGIR signaling. Serum PGI2 levels are decreased in CD patients with stenosis and are negatively correlated with disease duration. The PTGIR agonist inhibited the profibrotic function of YAP/TAZ in intestinal fibroblasts *in vitro* and reversed intestinal fibrosis *in vivo*.

**Conclusions:**

The antifibrotic effects of PGI2-PTGIR signaling are impaired in CD. Restoring PGI2-PTGIR signaling is a pharmacologically tractable and cell-selective approach to targeting YAP/TAZ via PTGIR, which reverses intestinal fibrosis.

## Graphical Abstract

**Graphical Abstract:**
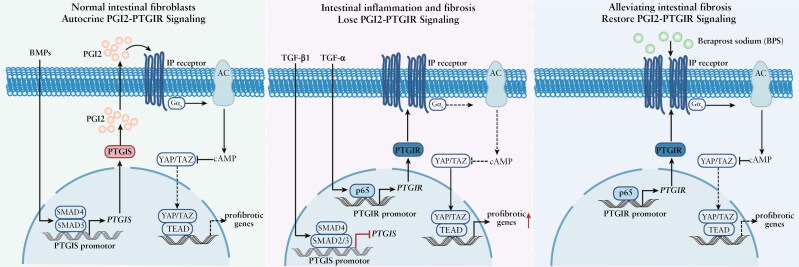

## 1. Introduction

Structural fibrosis and the development of intestinal obstruction are severe complications of Crohn’s disease (CD), a major form of inflammatory bowel disease (IBD) caused by extracellular matrix (ECM) deposition on the mucosa and submucosa due to chronic inflammation and recurrent healing of the mucosa.^1,2^ Over 50% of CD patients undergo surgery due to stenotic complications within a 10-year disease duration.^3–5^ However, surgery does not prevent fibrogenesis or stenotic recurrence. Currently, anti-inflammatory treatments, such as aminosalicylate, steroids, and immunosuppressants, are widely used for the clinical treatment of IBD.^6,7^ Although inflammation can initiate fibrogenesis and alleviate fibrosis by eliminating harmful stimuli and inhibiting inflammatory responses,^8,9^ anti-inflammatory therapies fail to effectively prevent intestinal fibrosis and obstruction progression. To date, no clinically effective antifibrotic therapy is available for intestinal fibrosis in patients with CD. Moreover, endoscopic and environmental factors, such as the early use of azathioprine, disease activity, smoking, and tissue/serum biomarkers, have been assessed to predict fibrostenotic CD. However, current evidence shows limited capacity of these factors to predict the risk of developing fibrostenotic complications in CD patients.^10^ There is still an urgent need in clinical practice to identify reliable predictors of fibrostenotic development.

Although the etiology of intestinal fibrosis in CD has not been fully elucidated, previous studies have revealed that the activation of fibroblasts by various cell types, cellular transformations, and soluble mediators lead to the production and secretion of collagen, cytokines, and other ECM components, which are crucial in the initiation and progression of intestinal fibrosis.^11–13^ Under physiological conditions, the normal structure and physiological function of fibroblasts in a stationary state are necessary to maintain homeostasis in the intestinal microenvironment.^12^ However, under pathological conditions, fibroblasts are activated by various signaling pathways and differentiate into myofibroblasts, which express smooth muscle actin (α-SMA) and induce a profibrotic phenotype that includes hyperproliferation and matrix deposition.^12^ Fibroblast activation of Yes-associated protein (YAP) and transcriptional coactivator with PDZ-binding motifs (TAZ), the transcriptional effectors of the Hippo signaling pathway, has emerged as a key step in organ fibrogenesis.^14,15^ Our recent study revealed the activation and profibrotic function of YAP/TAZ in CD-related intestinal fibrosis.^16^ Activation of YAP/TAZ in fibroblasts promotes cell proliferation.^16^ In addition, fibroblastic activation of YAP/TAZ can act in coordination with TGF-β signaling pathway to enhance the transcription of myofibroblast markers (eg, *ACTA2*) and ECM-related genes (eg,*COL1A1, CTGF, FN1*).^16^ However, systematic inhibition of YAP/TAZ exacerbates intestinal injury and inflammation in mice with colitis.^16^ Promoting mucosal healing is a promising therapeutic strategy in IBD.^17^ Since YAP/TAZ is required for LGR5^+^ intestinal stem cells to maintain intestinal homeostasis and plays a vital role in revival stem cells for intestinal regeneration after tissue injury, global YAP/TAZ inhibition can lead to dysregulated YAP/TAZ function in intestinal epithelial cells (IECs) and impaired mucosal healing capacity.^18–20^ Therefore, pharmacological approaches selectively targeting YAP/TAZ inhibition in intestinal fibroblasts but not IECs are needed to combat fibrotic progression in the intestines.

G protein-coupled receptors (GPCRs) are a highly diverse class of membrane receptors comprising more than 800 receptors and have been exploited as drug targets for numerous diseases, including organ fibrosis.^21^ In the past decade, GPCRs have been identified as important modulators of YAP/TAZ in multiple contexts; Gα_12/13_-, Gα_q/11_-, and Gα_i/o_-coupled receptors activate YAP/TAZ, whereas Gα_s_-coupled receptors inhibit YAP/TAZ nuclear localization and activity via increased cyclic adenosine monophosphate (cAMP).^21–23^ Gα_i/o_, Gα_q/11_, and Gα_12/13_ are profibrotic regulators, whose activation in fibroblasts can increase YAP/TAZ activity to drive fibrosis.^24^ Gα_s_ is an antifibrotic fibroblast modulator, and the diverse cell-type expression patterns of Gα_s_-coupled GPCRs suggest the possibility of specifically inhibiting YAP/TAZ in fibroblasts. Ga_s_-coupled dopamine receptor D1 (DRD1) was recently identified as a fibroblast-specific target for the treatment of pulmonary fibrosis and hepatic fibrosis through selective YAP/TAZ inhibition.^25^ DRD1 agonists have been shown to reverse lung and liver fibrosis via the inhibition of YAP/TAZ in fibroblasts. However, DRD1 is not expressed in intestinal fibroblasts, which prompted us to screen for Gα_s_-coupled GPCRs expressed in intestinal fibroblasts that are absent in IECs because of the selective inhibition of YAP/TAZ activity in intestinal fibroblasts.

The prostanoid prostacyclin, also referred to as prostaglandin I2 (PGI2), plays a vital physiologic role in the vasculature system and is widely implicated in cardiovascular, pulmonary, and renal diseases. PGI2 functions as a ligand of the Gα_s_-coupled prostacyclin/prostaglandin I2 receptor (PTGIR) and acts as a potent inhibitor of platelet aggregation and a dilator of vascular smooth muscle. Beraprost sodium (BPS) is an oral prostacyclin analog that is used as a pharmaceutical drug for the treatment of pulmonary hypertension. In addition to its important role in the vasculature system, the prostacyclin receptor was recently found to be expressed in pulmonary fibroblasts, whose activation inhibits the proliferation of fibroblasts by suppressing the platelet-derived growth factor-activated ERK pathway.^26^ The activation of the prostacyclin receptor by its agonists has been shown to have antifibrotic effects on lung and renal fibrosis.^26–29^ In IBD patient biopsies, PGI2 levels are significantly decreased in the mucosa, and PGI2 treatment alleviates colitis in dextran sulfate sodium (DSS)-induced colitis mice.^30^ Moreover, genome-wide analysis of DNA methylation revealed downregulated expression of prostaglandin I2 synthase (PTGIS) and hypermethylation of the PTGIS gene in patients with fibrotic CD. However, the dysregulation of PGI2-PTGIR signaling and its therapeutic potential in intestinal fibrosis have yet to be explored.

In this study, we report dysregulated PGI2-PTGIR signaling in CD-related intestinal fibrosis. PTGIS expression is downregulated in TGF-β-activated intestinal fibroblasts and fibroblasts derived from fibrostenic intestines. Serum levels of PGI2 are reduced in CD patients with intestinal obstruction, which is correlated with disease duration. PTGIR is absent in IECs and highly expressed in fibroblasts derived from stenotic intestines, and its expression can be induced by the inflammatory cytokine tumor necrosis factor α (TNF-α). Activating PGI2-PTGIR signaling inhibits YAP/TAZ activity in intestinal fibroblasts and alleviates fibrosis in a chronic colitis model mice. Therefore, activating PGI2-PTGIR signaling could be an effective strategy for specific inhibition of YAP/TAZ in intestinal fibroblasts to prevent the development of intestinal fibrosis in CD patients.

## 2. Methods

### 2.1. Study population and patient specimens

A retrospective cohort of 118 eligible CD patients was used in this study. The CD patients were enrolled and followed up from 2023 March to 2024 March in the Department of Gastroenterology, the Tenth People’s Hospital affiliated with Tongji University, and the Department of Gastroenterology and Department of Colorectal and Anal Surgery, Xinhua Hospital affiliated with Shanghai Jiaotong University School of Medicine. The diagnosis of CD was based on clinical symptoms, endoscopy, and histologic criteria. A fibrostenotic intestine was defined according to the Montreal classification,^31^ and fibrotic stenosis was confirmed by medical imaging, endoscopy, and/or surgery. The serum samples used in this study were collected from healthy volunteers and CD patients at the latest follow-up. For CD patients underwent fibrotic intestine removal surgery, serum samples were collected before surgery. Since the small intestine, especially the terminal ileum, is the most common site involved in intestinal fibrosis in Crohn’s disease,^32^ the tissue samples used in this study were from the small intestine. The area of stenosis was identified by 2 independent pathologists via macroscopic evaluation and was confirmed by histology. The study was approved by the Ethics Committee of Xinhua Hospital (No. XHEC-NSFC-2020-167).

### 2.2. Fibroblast isolation

Human fibroblasts were isolated from stenotic and nonstenotic regions of intestinal samples from CD patients.^33^ Briefly, fresh intestinal tissues were washed for 30 min at 37° C in phosphate-buffered saline (PBS) supplemented with high-concentration penicillin (500 U/mL)/streptomycin (500 mg/mL) while shaking and then digested with an enzyme mixture containing DNase I (1000 U/mL, Sigma‒Aldrich, USA), collagenase IV (0.1 U/mL, Sigma-Aldrich, USA), and dispase II (1.5 U/mL, Sigma-Aldrich, USA) until the tissues were fully dissolved. The cells were cultivated with FGM-2 Fibroblast Growth Medium-2 (Lonza, Basel, Switzerland).^16^ The morphology of typical spindle-forming fibroblasts could be observed after approximately 2–3 weeks. The attached cells were subsequently verified as fibroblasts by immunofluorescence (IF) staining of vimentin before subsequent analysis. Primary fibroblasts at passage 7 were used in this study.

### 2.3. Immunoblotting

Western blotting was performed as previously described.^16^ Fibroblasts were lysed with 1% NP40 lysis buffer (Sangon Biotech Co., Ltd., China) supplemented with NaF (Sangon Biotech Co., Ltd., China), Na_3_VO_4_ (Sangon Biotech Co., Ltd., China), and a protease inhibitor cocktail.^16^ Equal amounts of total protein were separated via sodium dodecyl sulfate‒polyacrylamide gel electrophoresis and transferred to nitrocellulose membranes (Millipore, USA). The membranes were incubated with specific antibodies at 4 °C overnight after being blocked with 5% nonfat milk for 1 h at room temperature. After incubation with a horseradish peroxidase-labeled secondary antibody (Beyotime, China), the blots were visualized via chemiluminescence (Millipore, USA). The antibodies used are listed in Table S4.

### 2.4. ELISA

A human PGI2 ELISA Kit (YoBiBiotech Co., Ltd., China) was used in this study according to the manufacturer’s instructions. In brief, 100 μL of the assay dilution and 50 μL of sample were added to each well. The plate was incubated with horizontal shaking for 2 h at room temperature. The plates were then washed 3 times with washing buffer. Then, the conjugate solution was added to each well and incubated for 1 h on a shaker. After incubation, the plates were washed and then incubated for 30 min after streptavidin-HRP1 was added to each well. Two hundred microliters of the substrate solution was added to each well after the plate was washed 3 times. The plate was incubated in the dark for 30 min. Finally, the absorbance was read at a wavelength of 450 nm 30 min after the addition of the stop solution. The PGI2 levels were quantified according to the standard curve.

For simultaneous analysis of PGI2 and PTGIS levels in intestinal tissues, tissue specimens (around 0.1 g) were homogenized in 1 mL of PBS with protease inhibitors by ultrasonication, followed by centrifugation at 3000 g for 20 min at 4 °C to obtain supernatant fractions. PGI2 level in supernatants was measured by ELISA, which was normalized to the protein concentration measured by BCA Protein assay kit. PTGIS expressions were examined by Western blot and semiquantitative analysis of PTGIS protein levels was performed.

### 2.5. Real-time quantitative polymerase chain reaction

Paired human primary intestinal fibroblast samples and fibroblasts with or without small interfering RNA (siRNA) transfection treated with TGF-β1 and Beraprost sodium (BPS) were used to detect the expression levels of profibrotic genes and YAP/TAZ downstream target genes. The cells used in the real-time quantitative polymerase chain reaction (RT‒qPCR) experiment were seeded at low density in 6 cm dishes. Total RNA was extracted via TRIzol reagent (Vazyme Co., Ltd., China) and reverse transcribed into cDNA using a HiScript IV RT SuperMix for qPCR kit (Vazyme Co., Ltd., China). Universal SYBR qPCR Master Mix (Vazyme Co., Ltd., China) and an Applied Biosystems 7500 Fast Real-Time PCR system (Foster City, CA, USA) were used for qPCR. Relative mRNA expression was evaluated via the 2^−ΔΔCt^ method and normalized to the expression of *GAPDH*. All of the experiments were performed in triplicate. The sequences of the PCR primers used in this study are listed in Table S5.

### 2.6. Small interfering RNA transfection

The siRNAs targeting human PTGIR and PTGIS are listed in Table S5. The siRNAs and nonspecific control siRNA duplexes were synthesized by Shanghai Jima Gene Co., Ltd. (Shanghai, China). Human primary intestinal fibroblasts were seeded at approximately 2 × 10^5^ cells/well, and the next day, the fibroblasts were transfected with siRNA using serum-free Opti-Minimal Essential Medium solution (Gibco, USA) with Lipofectamine RNAiMAX (Invitrogen, USA) for 48 h before RNA and protein extraction.

### 2.7. Immunofluorescence staining

Immunofluorescence staining was performed as previously described.^16^ In brief, 4% paraformaldehyde was used to fix cells grown on cover slides for 30 min at room temperature, after which they were permeabilized by exposure to 0.1% Triton X-100 for 10 min. After being blocked in 3% bovine serum albumin (BSA) for 30 min at room temperature, the cells were incubated with primary antibodies (1:100 anti-α-SMA and 1:100 anti-YAP) in 1% BSA overnight at 4 °C. After being washed with PBS, the cells were incubated with Cy3-labeled goat anti-rabbit IgG and Alexa Fluor 488-conjugated goat anti-mouse IgG in the dark for 1 h. Finally, the cells were counterstained with 5 μg/mL 4′,6-diamidino-2-phenylindole and analyzed using fluorescence microscopy (OLYMPUS, Japan). YAP subcellular localization was quantified by comparing the immunofluorescent signal intensity in nucleus and cytoplasm. “N” represents the nuclear immunofluorescent signal intensity and “C” indicates cytoplasmic immunofluorescent signal intensity. “N>C” represents nuclear accumulation of YAP and “N<C” indicates cytoplasmic retention of YAP. “N=C” represents the homogeneously diffused localization of YAP in both nucleus and cytoplasm. Hundred cells in at least 5 randomly selected low-magnification fields per group were analyzed to ensure statistical validity.

### 2.8. Cut&Run

Cut&Run assays were carried out via the Hyperactive pG-MNase CUT&RUN Assay Kit for PCR/qPCR (Vazyme Co., Ltd., China). Briefly, 2 × 10^6^ living cells were prepared according to the manufacturer’s instructions, captured with ConA beads and incubated with primary antibodies overnight at 4 °C. After the unbound antibody was washed away, pG-MNase enzyme was added at a 1:100 ratio, and the mixture was incubated for 1 h at 4 °C. The nuclei were washed again, and CaCl_2_ was added to activate the pG-MNase enzyme. The reaction was carried out for 4 h at 4 °C and stopped by the addition of an equal volume of stop buffer. The protein‒DNA complex was released by centrifugation and then incubated with GDP buffer. DNA was extracted via ethanol precipitation and collected via columns. The antibodies used are listed in Table S4.

### 2.9. Luciferase reporter assay

The WT and NF-κB binding site mutant PTGIR promoters (−858 bp~ + 45 bp) and the PTGIS promoter (−1179 bp~+376 bp) were inserted into the pGL3 luciferase reporter vector. 293T cells were plated on 24-well plates and transiently cotransfected with the PTGIR luciferase promoter or PTGIS luciferase promoter and the indicated plasmids for 24 h. For TNF-α treatment, 293T cells were transfected with the PTGIR luciferase promoter and Renilla luciferase reporter plasmids for 6 h and then incubated with TNF-α for 18 h. The luciferase activity was measured via a dual luciferase reporter assay (Promega, USA) and normalized to the activity of Renilla luciferase.

### 2.10. Dextran sulfate sodium (DSS)-induced acute and chronic colitis models

The Animal Care and Welfare Committee of Xinhua Hospital approved all of the animal experiments in this study. Six-week-old specific pathogen-free (SPF) C57BL/6 male mice (weighing 20 g each) were purchased from the Experimental Animal Center of the Chinese Academy of Sciences (Shanghai, China) and maintained in an SPF animal experiment room (constant temperature of 20 ± 3 °C with 55% ± 10% humidity and a 12 h light/dark cycle). Acute colitis was induced by the addition of 3% DSS to the drinking water for 7 days.^34^ Chronic colitis was induced by treatment with 2% DSS for 7 days followed by 2 weeks of tap water for a total of 3 cycles.^34^ For BPS treatment in the chronic colitis murine model, the mice were randomly assigned to the control or DSS-induced groups. In the last cycle of colitis induction, the mice were gavaged with solvent or BPS (0.6 mg/kg/day).^35^ Mice were monitored for body weight, activity, and certain conditions, such as diarrhea and bloody stools. Mice that died before completion of the 9-week experimental protocol were excluded from further data analysis. All living mice were euthanized after the acute/chronic inflammation models were established and after the experiments were performed. No anesthetic was used in the animal experiments. Investigators who performed endpoint analyses were blinded to group allocation.

### 2.11. Immunohistochemistry

Immunohistochemistry (IHC) analysis was performed as described in a previous study.^16^ In brief, paraffin-embedded tissues were deparaffinized in xylene and rehydrated with descending concentrations of ethanol. The antigens were retrieved in citrate buffer by heating, and 3% hydrogen peroxide (Sangon Biotech Co., Ltd., China) and 5% goat serum (Beyotime Biotechnology, China) were used for blocking. Specific antibodies were incubated with the samples at 4 °C overnight, followed by incubation with an HRP-labeled secondary antibody for 1 h at room temperature. The chromogen diaminobenzidine was used for the chromogenic reaction. The quantitative immunohistochemical score was evaluated by densitometry. Briefly, IHC images underwent color deconvolution to isolate DAB chromogenic signals from HE counterstaining. The grayscale images were transformed to extract optical density values. Positive threshold was equally set to all samples and integrated density value of each sample was calculated and normalized to the mean value of control group for statistical analysis.

### 2.12. Histopathologic analysis and evaluation of inflammation and fibrosis

For histopathological evaluation, paraffin-embedded intestinal sections were stained with hematoxylin–eosin (H&E) and Masson’s trichrome. The inflammation score in the acute and chronic colitis models was determined by H&E staining on the basis of tissue damage (0 = none, 1 = isolated and focal epithelial injury, 2 = mucosal erosion and ulcers, and 3 = extensive damage to the entire intestinal wall) and inflammatory cell infiltration (0 = few, 1 = increased neutrophils in the mucosa, 2 = inflammatory cell mass in the mucosa and submucosa, and 3 = infiltration of inflammatory cells in all layers), as previously reported.^34^ Fibrotic changes were indicated by the fibrosis score determined from Masson’s trichrome staining. Fibrosis in the animal models was quantified via a combined score of fibrosis severity (0 = none, 1 = increased ECM deposition in the mucosa, 2 = increased ECM deposition in the submucosa, 3 = thickening of the muscularis mucosae in addition to ECM deposition in the submucosa, 4 = thickening of the muscularis propria in addition to ECM deposition in the submucosa, and 5 = ECM deposition in the serosa layers) and circularity (1 = 0%–25%, 2 = 25%-50%, 3 = 50%-75%, and 4 = 75%-100%).^36^ The data were evaluated by 2 independent pathologists.

### 2.13. Statistical analysis

GraphPad Prism 8 software (GraphPad Software, San Diego, CA, USA) was used for the statistical analyses. The data are expressed as mean ± standard deviation (S.D.). Student’s *t* test was used to compare differences between 2 groups. Paired Student’s *t* test was performed to assess the statistical significance of differential *PTGIR* and *PTGIS* mRNA expression in 6 pairs of CD-fibroblasts and adjacent normal fibroblasts. One-way analysis of variance (ANOVA) with Dunnett’s multiple comparison test was used to assess the statistical significance for the experiments with > 2 independent groups. Scoring data were analyzed by using a Mann–Whitney *U* test. All of the statistical tests were 2-sided, and a *P* value of < .05 was considered statistically significant.

## 3. Results

### 3.1. PTGIR is selectively expressed and upregulated in fibroblasts from the stenotic intestine

**Figure 1.**
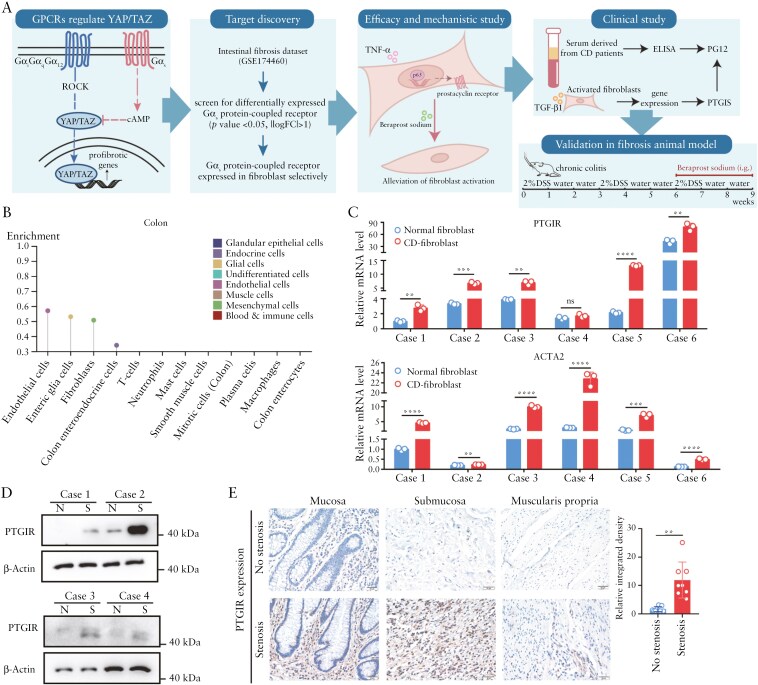
**Fibroblast PTGIR expression is increased in the stenotic intestine.** (A) A schematic diagram of the identification, functional, and clinical validation of PGI2-PTGIR signaling in this study. (B) PTGIR expression patterns in different tissue cell types. Data were extracted from the Human Protein Atlas. (C) *PTGIR* and *ACTA2* expression was detected in fibroblasts isolated from stenotic and nonstenotic areas of intestines from CD patients via qPCR. (D) Western blot analysis of PTGIR expression in 4 pairs of intestinal fibroblasts derived from stenotic and nonstenotic tissues. (E) Representative images of immunohistochemical staining and semiquantitative analysis of PTGIR expression in different layers of stenotic and nonstenotic intestinal tissues from CD patients (*n* = 8). Scale bar = 20 μm. In all cases, the bars in the graphs represent the mean ± S.D. Statistical analyses were performed via Student’s *t* test (C) and the Mann‒Whitney *U* test (E). Significant differences are shown by ^**^P < .01, ^***^P < .001, and ^****^P < .0001.

To identify the Gα_s_-coupled GPCR that specifically targets YAP/TAZ in fibroblasts for use in antifibrotic therapy in the intestine, we screened GPCRs based on 3 principles: it is specifically expressed in intestinal fibroblasts but not in IECs; the expression of the GPCR is increased in fibroblasts from stenotic tissues; and the agonist of the GPCR is available in the clinic (Figure 1A). Therefore, we reanalyzed the gene expression profiles of primary human intestinal fibroblasts derived from intestinal stenotic and paired nonstenotic tissues from CD patients (GSE174460),^16^ focusing on the GPCRome. Among the Gα_s_-coupled receptors, the prostacyclin receptor (PTGIR) was a promising candidate. PTGIR was strongly enriched in intestinal fibroblasts and endothelial cells but was barely expressed in IECs (Figure 1B).^37^ We first confirmed the expression of PTGIR in fibroblasts derived from stenotic and nonstenotic intestines via RT-qPCR. A significant increase in *PTGIR* mRNA levels was observed in fibroblasts from stenotic intestines, along with upregulation of the fibrotic marker gene *ACTA2* (Figure 1C). Western blot analysis also revealed that the protein level of PTGIR was increased in intestinal fibroblasts from stenotic patients (Figure 1D). Moreover, IHC analysis of intestinal stenotic and paired nonstenotic tissues revealed that PTGIR was specifically expressed in the intestinal stroma and that PTGIR expression was upregulated in the stroma of stenotic intestinal tissue (Figure 1E). Taken together, these data indicate that PTGIR is selectively highly expressed in fibroblasts from the stenotic intestine and is a potential target for the specific inhibition of YAP/TAZ in intestinal fibroblasts.

### 3.2. PTGIR agonism reverses profibrotic phenotypes by interfering with YAP/TAZ in intestinal fibroblasts

**Figure 2.**
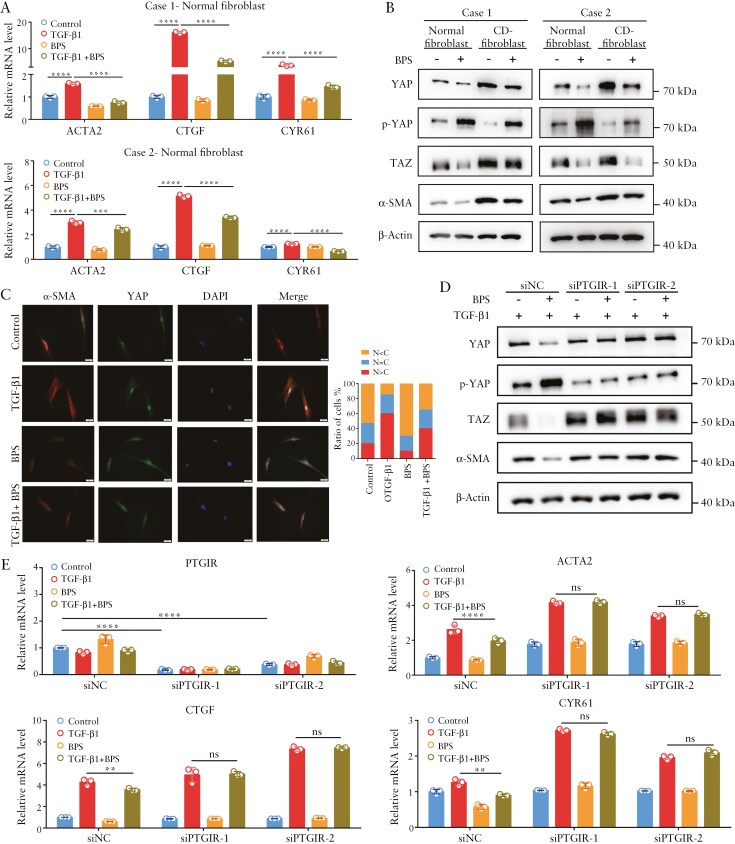
**PTGIR agonism reverses profibrotic phenotypes by interfering with YAP/TAZ.** (A) The effects of the PTGIR agonist BPS on *ACTA2* and the target genes of YAP/TAZ in intestinal fibroblasts treated with TGF-β1 were evaluated by qPCR. (B) The effects of the PTGIR agonist BPS on the expression of the YAP/TAZ proteins were analyzed via Western blotting. (C) Representative images of immunofluorescence staining showing the effect of the PTGIR agonist on YAP localization. Scale bar = 20 μm. (D) Intestinal fibroblasts were transfected with *PTGIR* siRNA for 2 days before the effect of the PTGIR agonist on YAP/TAZ expression was evaluated via Western blotting. (E) The effects of the PTGIR agonist on the mRNA levels of *ACTA2* and YAP/TAZ target genes in intestinal fibroblasts with *PTGIR* knockdown were analyzed via qPCR. In all cases, the bars in the graphs represent the mean ± S.D. Statistical analyses were performed via one-way ANOVA. Significant differences are shown by ^**^P < .01, ^***^P < .001, and ^****^P < .0001.

Beraprost sodium (BPS) is an oral PGI2 analog that functions as a PTGIR agonist. To explore whether PTGIR activation inhibited the activation of YAP/TAZ and the expression of profibrotic genes induced by TGF-β, human primary fibroblasts isolated from the nonstenotic intestine were treated with TGF-β with or without BPS. As shown in Figure 2A, the upregulated mRNA levels of the profibrotic gene *ACTA2* and the classic YAP/TAZ target genes *CTGF* and *CYR61* induced by TGF-β were significantly diminished by the PTGIR agonist BPS. Similar results were observed via Western blot analysis, which revealed that BPS decreased the protein levels of YAP/TAZ and α-SMA but increased the level of p-Ser127 (YAP) in both normal fibroblasts and fibroblasts isolated from the fibrostenotic intestines (Figure 2B). Consistent with the above results, IF staining revealed that the nuclear translocation of YAP induced by TGF-β1 was abrogated in intestinal fibroblasts treated with BPS (Figure 2C). To further strengthen the receptor specificity of the effect of BPS on YAP/TAZ, PTGIR knockdown in intestinal fibroblasts was performed before BPS treatment. Interestingly, we detected increased protein levels of TAZ and decreased levels of p-S127 (YAP) in PTGIR-knockdown fibroblasts, indicating the inhibitory effect of endogenous PTGIR signaling on YAP/TAZ (Figure 2D). Moreover, BPS treatment had no effect on the protein levels of YAP/TAZ or p-S127 (YAP) (Figure 2D). Similar results were observed via IF staining of YAP and qPCR analysis of *CTGF, CYR61*, and *ACTA2* in PTGIR-knockdown fibroblasts (Figures 2E and S1A). Taken together, these data demonstrate that PTGIR agonists suppress the profibrotic phenotypes of intestinal fibroblasts, likely through inhibiting YAP/TAZ activity.

### 3.3. NF-κB promotes the transcriptional activation of PTGIR in intestinal fibroblasts in response to TNF-α

**Figure 3.**
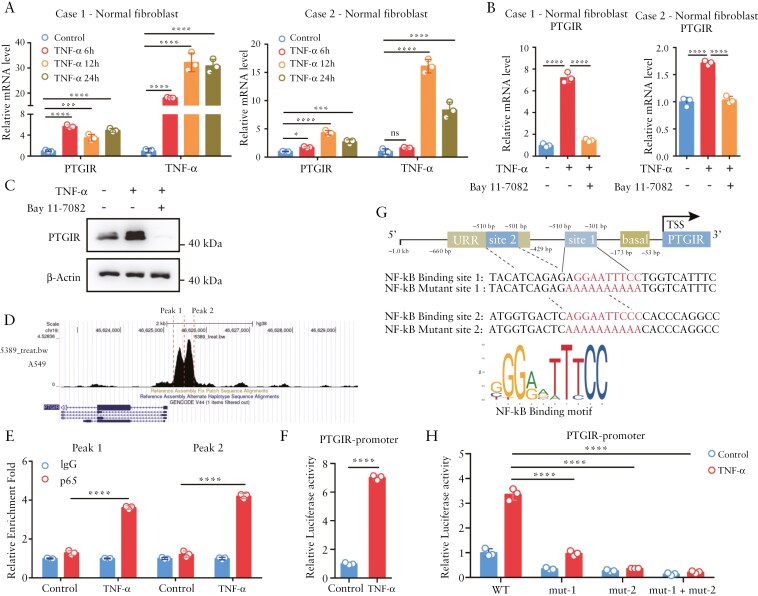
**p65 directly activates *PTGIR* gene transcription in intestinal fibroblasts stimulated with TNF-α.** (A) The mRNA levels of *PTGIR* were detected via qPCR in intestinal fibroblasts stimulated with TNF-α (10 ng/mL) for the indicated times. (B) *PTGIR* expression was detected via qPCR in intestinal fibroblasts pretreated with Bay 11-7082 (30 µM) for 1 h and subsequently stimulated with TNF-α for 4 h. (C) Western blot analysis of PTGIR expression in intestinal fibroblasts treated with TNF-α (10 ng/mL, 12 h) and Bay 11-7082 (30 µM, 4 h). (D) Representative sequencing tracks of the p65 ChIP-seq data at the *PTGIR* genomic locus. Data were extracted from the Cistrome DB database. (E) CUT&RUN analysis of p65 binding to the *PTGIR* promoter in intestinal fibroblasts stimulated with TNF-α (10 ng/mL) for 24 h. (F) Luciferase reporter analysis of the *PTGIR* promoter reporter stimulated by TNF-α. HEK-293T cells were transfected with the *PTGIR* promoter for 24 h and stimulated with TNF-α (10 ng/mL) for 24 h before luciferase activity was detected. (G) Schematic depiction of the *PTGIR* gene locus showing potential NF-κB binding sites and the corresponding DNA sequence of the mutant *PTGIR* luciferase reporter. The known basal core promoter and upstream repressor region of the *PTGIR* gene are indicated. (H) Luciferase reporter assay of *PTGIR* WT and mutant promoter reporters in HEK-293T cells stimulated with TNF-α (10 ng/mL) for 24 h. In all cases, the bars in the graphs represent the mean ± S.D. Statistical analyses were performed via one-way ANOVA (A, B, E, H) and Student’s *t* test (F). Significant differences are shown by ^*^P < .05, ^**^P < .01, ^***^P < .001, and ^****^P < .0001.

The transcription of PTGIR can be increased in response to cellular differentiation, estrogen, and low serum cholesterol in the vasculature.^38^ To explore the underlying mechanism of upregulated PTGIR expression in the fibrostenotic intestine, we examined the effects of TGF-β, the master regulator of fibrosis, and TNF-α, the key proinflammatory cytokine in IBD, on PTGIR transcription in primary intestinal fibroblasts. Intriguingly, we found that *PTGIR* mRNA levels were significantly increased in primary intestinal fibroblasts stimulated with TNF-α (Figure 3A) but not in those stimulated with TGF-β (data not shown). Not surprisingly, PTGIR induction by TNF-α in intestinal fibroblasts was abrogated by the NF-κB inhibitor Bay 11-7082 at both the mRNA and protein levels (Figure 3B and C).

Next, we sought to determine whether NF-κB directly regulates PTGIR gene transcription. The publicly available ChIP-seq dataset of the p65 subunit revealed that p65 could bind to the promoter region of PTGIR in lung epithelial cells (Figure 3D) (http://cistrome.org/db).^39^ Owing to the limited number of primary fibroblasts and the low cell requirements for Cut & Run assays, we performed Cut & Run analysis and confirmed that p65 bound to 2 similar promoter regions (peak 1 and peak 2) of PTGIR in intestinal fibroblasts (Figure 3E). We then generated a luciferase reporter of the PTGIR promoter and observed that TNF-α dramatically increased PTGIR promoter activity (Figure 3F). Bioinformatic analysis further revealed that 2 NF-κB binding sites were located in these 2 PTGIR promoter regions (Figure 3G). Individual mutation of the NF-κB binding sites not only led to downregulation of the basal activity of the PTGIR promoter but also largely abolished the increase in PTGIR promoter activity induced by TNF-α treatment (Figure 3H). Overall, these data demonstrate that NF-κB can directly modulate PTGIR gene transcription and that the upregulated PTGIR expression in the stroma is likely due to the inflammatory microenvironment during the progression of IBD.

### 3.4. Decreased expression of PTGIS in stenotic intestines and reduced serum PGI2 are associated with the duration of CD

**Figure 4.**
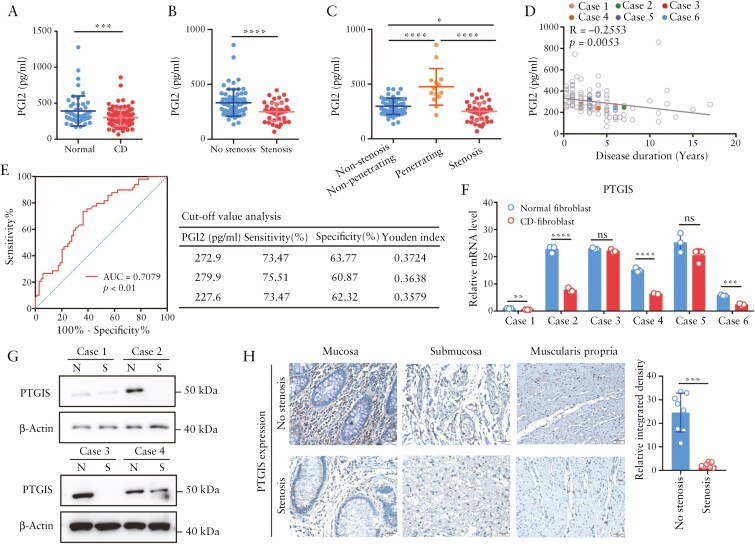
**Downregulation of PGI2 and PTGIS in patients with CD with stenosis.** (A) PGI2 levels in the serum of healthy controls and patients with CD were detected via ELISA. (B) PGI2 levels in the serum of stenotic and nonstenotic CD patients were compared. (C) Subgroup analysis of serum PGI2 levels in CD patients with different disease behaviors. (D) Pearson correlation analysis of the serum PGI2 levels and disease duration of CD patients (*n* = 118). (E) Receiver operating characteristic curve of the PGI2 level in the serum for the prediction of stenosis in CD patients. The Youden index was used to analyze the cutoff value of the PGI2 level in serum, and the maximum Youden index was 0.3724, with a sensitivity of 73.47% and specificity of 63.77% at the cutoff value of 272.9 pg/mL. (F) PTGIS expression was detected in fibroblasts isolated from stenotic and nonstenotic areas of intestines from CD patients via qPCR. (G) Western blot analysis of PTGIR expression in 4 paired fibroblasts isolated from stenotic and nonstenotic areas of intestines from CD patients. (H) Representative images of immunohistochemical staining of PTGIS protein in different layers of stenotic and nonstenotic intestinal tissue from CD patients. A semiquantitative immunohistochemical score was used for statistical analysis (*n* = 8). In all cases, the bars in the graphs represent the mean ± S.D. Statistical analyses were performed via Student’s *t* test for (A-B, F), one-way ANOVA for (C), Pearson’s correlation for (D) and the Mann‒Whitney *U* test for (H). Significant differences are shown by ^*^P < .05, ^**^P < .01, ^***^P < .001, and ^****^P < .0001.

Prostacyclin is a member of the lipid mediator family known as prostanoids and is the main product of arachidonic acid metabolism formed by the large vessel endothelium. It is recognized as an important ligand for the prostacyclin receptor (IP receptor).^40^ Gα_s_-coupled GPCRs elicit antifibrotic signaling; however, the loss of Gα_s_-coupled GPCRs has been widely observed in multiple organ fibrosis. Thus, although PTGIR expression is upregulated in the fibrostenotic intestine, we speculated that PGI2-PTGIR signaling is impaired in intestinal fibroblasts. We first examined the serum level of PGI2 in a retrospective cohort consisting of 118 eligible CD patients and 50 healthy volunteers. The detailed demographic and clinical characteristics of the patients in the CD cohort are shown in Table S1. We found that the serum PGI2 level was significantly lower in CD patients than in healthy controls (Figure 4A). Notably, the serum PGI2 level in CD patients with stenosis was lower than that in those without stenosis (Figure 4B). Interestingly, CD patients with penetrating complications presented increased serum levels of PGI2, indicating dynamic variations in PGI2 levels during disease progression (Figure 4C).

Moreover, we analyzed the correlation between the serum PGI2 level and disease duration in patients with CD. Intriguingly, we observed that the serum PGI2 level in CD patients was negatively correlated with disease duration (*R* = −0.2552, *P* = .0053) (Figure 4D). We further assessed the clinical value of the serum PGI2 level. The serum PGI2 level had the most significant area under the ROC curve (AUC) of 0.71, with a sensitivity of 73.47%, specificity of 63.77%, and Youden index of 0.3724, at a cutoff value of 272.9 pg/mL (Figure 4E). The cohort was divided into a high PGI2 level group (PGI2 ≥ 272.9 pg/mL) and a low PGI2 level group (PGI2 < 272.9 pg/mL). On the basis of these criteria, 61 (51.7%) and 57 (48.3%) samples were categorized into low- and high-PGI2 groups, respectively. Similar to the results shown in Figure 4C, low serum PGI2 levels were significantly associated with the occurrence of intestinal obstruction (*P* < .001), whereas high serum PGI2 levels were correlated with fistulas (*P* = .002) (Table S2). Both univariate and multivariate logistic regression analyses revealed that the PGI2 level in the serum was significantly associated with intestinal stenosis (OR = 4.772, 95% CI = 1.981-11.494; *P* < .001) (Table S3).

Next, we examined the expression of PTGIS, the key enzyme that regulates PGI2 biosynthesis,^41^ in fibroblasts derived from stenotic and nonstenotic intestines. In 6 paired intestinal fibroblasts, significantly decreased *PTGIS* mRNA levels were detected in fibroblasts from stenotic intestines in 4 CD patients (Figure 4F). Western blot analysis further confirmed the downregulation of PTGIS protein expression in intestinal fibroblasts derived from stenotic intestines (Figure 4G). Moreover, decreased PTGIS expression in stenotic intestinal tissue was confirmed by IHC (Figure 4H). Furthermore, we simultaneously analyzed the PGI2 levels and PTGIS expressions in 13 stenotic intestinal tissues and paired nonstenotic tissues. Both expressions of PTGIS and PGI2 levels were decreased in stenotic intestines (Figure S2A and B). Importantly, the expression levels of PTGIS were positively correlated with the PGI2 levels in intestinal tissues (Figure S2C). Taken together, these data indicate that downregulated expression of PTGIS leads to decreased PGI2 synthesis, which might account for the impaired PGI2-PTGIR signaling in intestinal fibroblasts.

### 3.5. PTGIS inhibits YAP/TAZ activation through PGI2-PTGIR signaling in intestinal fibroblasts

**Figure 5.**
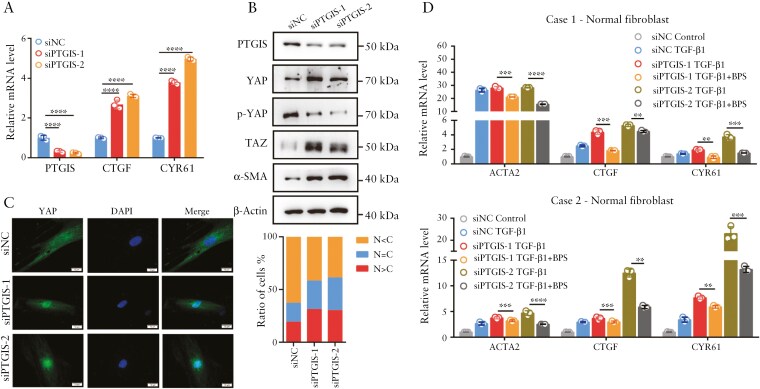
**PTGIS is a negative regulator of YAP/TAZ in intestinal fibroblasts.** (A) qPCR analysis of YAP/TAZ target genes in intestinal fibroblasts derived from stenotic intestines with *PTGIS* knockdown. (B) Western blot analysis of the effects of *PTGIS* knockdown on YAP/TAZ activation and α-SMA expression. (C) Representative images of immunofluorescence staining of YAP in intestinal fibroblasts derived from stenotic intestines with *PTGIS* knockdown. Scale bar = 20 μm. (D) The elevated mRNA levels of *ACTA2* and YAP/TAZ target genes in *PTGIS*-knockdown intestinal fibroblasts were reversed by the PTGIR agonist BPS. In all cases, the bars in the graphs represent the mean ± S.D. Statistical analyses were performed via one-way ANOVA. Significant differences are shown by ^**^P < .01, ^***^P < .001, and ^****^P < .0001.

Next, we examined whether PTGIS affects the activation of YAP/TAZ and the expression of profibrotic genes in intestinal fibroblasts. As expected, the knockdown of *PTGIS* by 2 independent siRNAs targeting *PTGIS* led to the upregulation of target genes of YAP/TAZ, including *CTGF* and *CYR61*, in intestinal fibroblasts (Figure 5A). Western blot analysis also revealed that the expression of α-SMA, YAP, and TAZ were increased, whereas that of p-S127 (YAP) was decreased upon *PTGIS* knockdown (Figure 5B). Consistently, the nuclear localization of YAP was enhanced in primary intestinal fibroblasts with *PTGIS* knockdown (Figure 5C).

Next, we explored whether the stimulating effect of *PTGIS* knockdown on YAP/TAZ activity and profibrotic genes could be due to impaired PGI2 synthesis and whether the activation of YAP/TAZ and the expression of profibrotic genes induced by *PTGIS* knockdown in activated intestinal fibroblasts could be reversed by the PTGIR agonist BPS. In intestinal fibroblasts activated with TGF-β, *PTGIS* knockdown still mildly to moderately promoted the transcription of the profibrotic and YAP/TAZ target genes (Figure 5D). Furthermore, the increased expression of *ACTA2*, *CTGF* and *CYR61* caused by *PTGIS* knockdown was reversed by BPS treatment (Figure 5D). Gαs-coupled receptors are known to act through cAMP-PKA to stimulate LATS kinases and YAP phosphorylation.^42^ Indeed, the increased protein levels of YAP/TAZ and decreased phosphorylated YAP levels induced by *PTGIS* knockdown in activated intestinal fibroblasts were accompanied by decreased activity of LATS kinase (indicated by p-LATS1 levels), which could also be reversed by the PTGIR agonist BPS (Figure S3). Meanwhile, the inhibitory effect of BPS on YAP/TAZ was largely abrogated by treatment of LATS kinase inhibitor VT02956 (Figure S3). These data suggest that PTGIS suppresses fibroblastic YAP/TAZ activation and intestinal fibrosis through the synthesis of PGI2 and autocrine PGI2-PTGIR signaling and subsequent LATS activation.

### 3.6. TGF-β-SMAD2/3 signaling antagonizes the transcriptional activation of PTGIS induced by BMP2/9-SMAD5 signaling in intestinal fibroblasts

**Figure 6.**
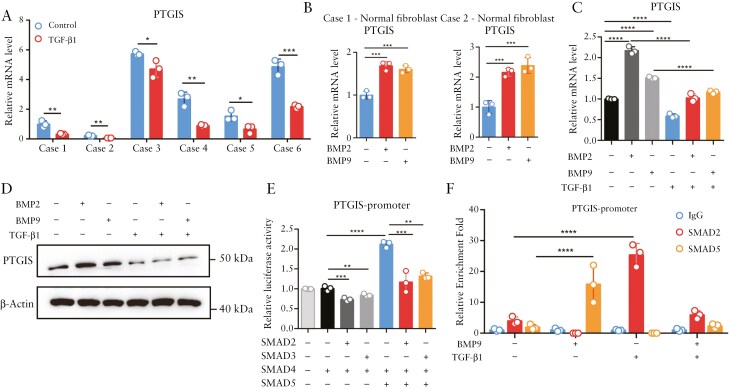
**TGF-β-SMAD2/3 counteracts the transcriptional activation of *PTGIS* by BMP2/9-SMAD5 in intestinal fibroblasts.** (A) qPCR analysis of the mRNA levels of *PTGIS* in normal intestinal fibroblasts treated with TGF-β1 (10 ng/mL, 24 h). (B) qPCR analysis of the mRNA levels of *PTGIS* in normal intestinal fibroblasts treated with BMP2 (10 ng/mL, 24 h) or BMP9 (10 ng/mL, 24 h). (C) qPCR analysis of *PTGIS* mRNA levels in normal intestinal fibroblasts treated with BMP2 (10 ng/mL, 24 h) or BMP9 (10 ng/mL, 24 h) alone or cotreated with TGF-β1 (10 ng/mL, 24 h). (D) Western blot analysis of PTGIS protein levels in normal intestinal fibroblasts treated with BMP2 (10 ng/mL, 24 h) or BMP9 (10 ng/mL, 24 h) alone or in combination with TGF-β1 (10 ng/mL, 24 h). (E) A luciferase reporter assay was used to analyze the effects of SMAD2/3 and SMAD5 on the transcriptional activity of the *PTGIS* promoter in HEK-293T cells. (F) CUT&RUN analysis of SMAD2 or SMAD5 binding to the *PTGIS* promoter in intestinal fibroblasts stimulated with TGF-β1 (10 ng/mL) and/or BMP9 (10 ng/mL) for 24 h. In all cases, the bars in the graphs represent the mean ± S.D. Statistical analysis was performed via Student’s t test for (A) and one-way ANOVA for (B-C, E-F). Significant differences are shown by ^*^P < .05, ^**^P < .01, ^***^P < .001, and ^****^P < .0001.

We further explored the underlying mechanism of the decreased expression of PTGIS in the fibrostenotic intestine. Following the steps of *PTGIR* transcription mechanistic studies, we assessed the effects of both TGF-β and TNF-α on *PTGIS* transcription in primary intestinal fibroblasts. In contrast to the transcriptional activation of *PTGIR* by inflammatory stimuli, the mRNA level of *PTGIS* was significantly reduced by TGF-β (Figure 6A) but not TNF-α (data not shown) in primary intestinal fibroblasts derived from 6 CD patients. Recently, bone morphogenetic protein 2 (BMP2) and bone morphogenetic protein 9 (BMP9), members of the transforming growth factor-beta (TGF-β) superfamily, have been revealed to play suppressive roles in organ fibrosis.^43–46^ Interestingly, in contrast to the inhibitory effect of TGF-β, *PTGIS* expression was significantly upregulated by BMP2 and BMP9 treatment (Figure 6B). Moreover, the enhancing effect of BMP2/9 on PTGIS expression in intestinal fibroblasts could be largely attenuated by TGF-β1 treatment, and vice versa, which implied the counter and competitive role of TGF-β and BMP2/9 in *PTGIS* transcription in intestinal fibroblasts (Figure 6C and D).

BMPs elicit the activation of SMAD1/5/8, whereas TGF-β elicits the activation of SMAD2/3.^47^ Thus, we constructed a luciferase reporter of the *PTGIS* promoter and detected the effects of SMAD2/3 and SMAD5 overexpression on the *PTGIS* promoter reporter. Consistent with the above results, co-overexpression of SMAD4 and SMAD5 activated the luciferase activity of the *PTGIS* reporter, and co-overexpression of SMAD4 and SMAD2/3 inhibited the *PTGIS* promoter (Figure 6E). In addition, the overexpression of SMAD2/3 largely diminished the activation of the *PTGIS* reporter induced by the overexpression of SMAD5 (Figure 6E). Similar results were observed in the Cut&Run analysis of the *PTGIS* promoter in primary intestinal fibroblasts (Figure 6F). TGF-β1 promoted SMAD2 binding to the PTGIS promoter region and reduced the binding of SMAD5 to the *PTGIS* promoter under basal and BMP9-stimulated conditions, and vice versa (Figure 6F). Taken together, these data demonstrate that BMP2/9 activates while TGF-β suppresses the gene transcription of *PTGIS* in intestinal fibroblasts, probably through competitive binding of SMAD5 and SMAD2/3 to the *PTGIS* promoter.

### 3.7. Fibroblastic inhibition of YAP via activating PGI2-PTGIR signaling relieves intestinal fibrosis in a chronic colitis murine model

Recent studies revealed the potential anti-inflammatory role of PTGIR agonists in an acute colitis murine model.^30^ We first assessed the effect of the PTGIR agonist BPS on YAP *in vivo* in an acute colitis murine model in which the mice were given drinking water containing 3% DSS for 7 days and then treated with BPS beginning on the second day of induction via oral gavage once per day (Figure S2A). Consistent with previous reports, DSS-induced mice subjected to BPS treatment presented alleviated colitis, manifested as a longer colon length, lower disease activity index, and histopathological inflammation scores and less CD3^+^ T-cell infiltration than did mice subjected to DSS induction alone (Figure S2B-E). Interestingly, despite analysis at the acute colitis stage, we still detected fibrotic histological manifestations in DSS-induced mice, which consisted of mild collagen deposition in the intestinal mucosal layer and submucosal layer, and these mild fibrotic manifestations were alleviated by BPS treatment (Figure S2F). Moreover, IHC analysis of YAP revealed that BPS treatment significantly reduced the stromal expression level of YAP in colon tissues (Figure S2G).

**Figure 7.**
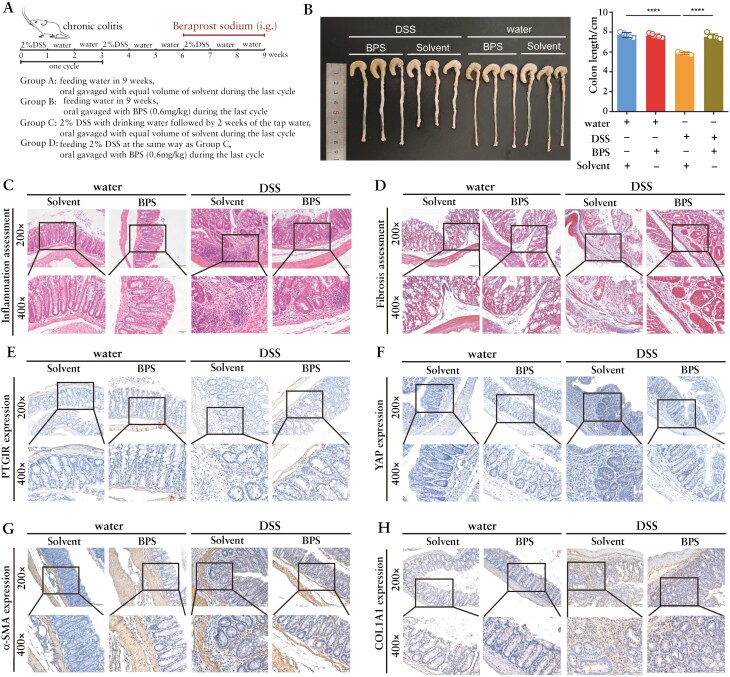
**PTGIR agonist reverses intestinal fibrosis in a chronic colitis murine model.** (A) Schematic diagram of PTGIR agonist treatment in a DSS-induced chronic colitis model. The mice were treated with 2% DSS to induce chronic inflammation and then with solvent or PTGIR agonist via oral gavage during the last cycle of colitis induction. (B-D) Colorectal length (B), representative images of hematoxylin‒eosin-stained sections (C) and Masson’s trichrome-stained sections (D) of the distal colon in mice subjected to different treatments. Scale bars = 50 μm (upper panels) and 20 μm (lower panels). (E-H) Representative immunohistochemical images showing PTGIR (E), YAP (F), α-SMA (G), and COL1A1 (H) expression in colonic tissues from mice subjected to different treatments. Scale bars = 50 μm (upper panels) and 20 μm (lower panels). In all cases, the bars in the graphs represent the mean ± S.D. Statistical analyses were performed via one-way ANOVA. Significant differences are shown by ^****^P < .0001.

Next, we explored whether BPS decreases YAP/TAZ expression and alleviates intestinal fibrosis in a chronic experimental colitis model. In the chronic colitis murine model, the mice were fed drinking water containing 2% DSS for 3 cycles and treated with BPS before the third cycle of DSS induction to further explore the antifibrotic effect of the PTGIR agonist *in vivo* (Figure 7A). Similar to the observations in the acute colitis model, chronic colitis model mice treated with BPS presented less severe manifestations than those treated with only DSS, as indicated by the longer length of the colon (Figure 7B). Moreover, H&E staining and Masson’s trichrome staining revealed that the colons from BPS-treated mice had lower pathological scores than those from DSS-induced mice did (Figures 7C and D and S3A and B). Consistently, IHC analysis revealed elevated expression of PTGIR in the stroma, which was downregulated in BPS-treated mice, probably due to alleviated intestinal inflammation in this colitis model (Figures 7E and S3C). Furthermore, treatment with the PTGIR agonist BPS significantly reduced the stromal expression of YAP and the profibrotic markers α-SMA and collagen Ⅰ (Figures 7F-H and S3D-F). Overall, these data indicate that the antifibrotic effect of the PTGIR agonist in both acute and chronic colitis murine models is correlated with reduced YAP stromal expression, which suggests that PGI2-PTGIR activation may prevent intestinal fibrosis by downregulating YAP.

## 4. Discussion

The wide expression and vital role of YAP/TAZ in mucosal repair and intestinal homeostasis highlight the necessity of developing a fibroblast-specific strategy for inhibiting YAP/TAZ function in intestinal fibrosis. In this study, we highlighted PTGIR, a Gα_s_ protein-coupled receptor, as a potential therapeutic target for intestinal fibrosis in patients with CD. PTGIR agonism suppresses the activation of intestinal fibroblasts by inhibiting YAP/TAZ activity. Importantly, PTGIR is absent in IECs, which provides a cell-selective approach that preserves the function of YAP/TAZ in IECs for mucosal healing.^18–20,48^ As the membrane receptor of PGI2/prostacyline, PTGIR is also expressed in endothelial cells and mediates the protective role of PGI2 in the vascular system.^38^ Intestinal fibroblasts/myofibroblasts can originate from various sources, including endothelial cells, via endothelial‒mesenchymal transition (EndoMT).^49^ A recent study reported that endothelial cAMP-dependent PKA-CREB signaling inhibits EndoMT by increasing BMPRII expression.^50,51^ Thus, we propose that PTGIR agonism could also exert antifibrotic effects via inflammation-induced EndoMT. In addition, PGI2 is required to prevent penetrating ulcers after colonic mucosal injury.^52^ PGI2 can inhibit intestinal epithelial permeability and apoptosis, alleviate colitis and prevent penetrating ulcers, probably through stimulating VEGF-dependent angiogenesis.^30^ Given the increased expression of PTGIR in fibrostenotic tissues and the multiple functions of PTGIR agonism in antifibrosis, anti-inflammatory, and mucosal healing, assessing the antifibrotic effects of PGI2 analogs in preventing intestinal fibrosis in clinical trials is promising.

Given the nature of Gα_s_ proteins as antifibrotic regulators, the loss of Gα_s_-coupled GPCRs is a common phenomenon in fibrosis. For example, the antifibrotic effect of prostaglandin E2 is limited by decreased expression of the prostaglandin receptor PTGER2 in idiopathic pulmonary fibrosis.^53,54^ Similarly, the absence of PTGIR in renal fibroblasts exacerbates the fibrotic process and impairs the antifibrotic effect of PGI2 analogs.^27^ In lung fibroblasts, TGF-β was found to induce HDAC-dependent transcriptional repression of multiple Gα_s_-coupled GPCR genes, including PTGIR.^55^ In contrast, we did not observe an effect of TGF-β on *PTGIR* transcription in intestinal fibroblasts, which highlights the difference between fibroblasts from different tissues.^56^ Furthermore, our study revealed that *PTGIR* expression is transcriptionally enhanced by TNF-α in intestinal fibroblasts; thus, the elevated PTGIR expression in fibrotic tissues is associated with the inflammatory environment in IBD. It is worth noting that anti-TNF monoclonal antibodies have not shown significant antifibrotic efficacy in clinic,^57^ which might be due to the downregulation of Gα_s_-coupled GPCRs by anti-TNF therapy, such as PTGIR. Notably, 1 NF-κB binding site is located in the upstream repressor region (URR) of the PTGIR promoter, which is bound by 2 trans-acting factors, C/EBPδ and PU.1.^58^ PMA induces PTGIR gene expression by alleviating the binding of C/EBPδ and enhancing the binding of PU.1 to the URR.^58^ Since inflammation-sensitive superenhancers are cobound by both PU.1 and NF-κB, we speculate that NF-κB binding might enhance the binding of PU.1 to the URR region and that NF-κB and PU.1 cooperate to activate *PTGIR* transcription upon TNF-α stimulation.

In addition to the transcriptional activation of *PTGIR*, the increased expression of PTGIR could also be due to the posttranscriptional regulation of PTGIR. A recent study reported that GPRC5B interacts with PTGIR and suppresses the membrane location of PTGIR in vascular smooth muscle cells. Notably, the mRNA level of *GPRC5B* was significantly decreased in intestinal fibroblasts from stenotic tissues (GSE174460).^16^ This could lead to enhanced membrane expression of PTGIR, which needs to be explored in future studies. Thus, the increased transcription and enhanced membrane expression of PTGIR might reinforce the response of pathogenic fibroblasts to PGI2 analogs, further supporting PTGIR as a promising target for treating intestinal fibrosis.

As mentioned above, the antifibrotic function of Gα_s_-coupled GPCRs is widely impaired in pathogenic fibroblasts. In the case of PTGIR, our study revealed that the expression of this receptor is upregulated in intestinal fibroblasts derived from stenotic tissues. However, PTGIS, the enzyme responsible for synthesizing the endogenous ligand PGI2, is downregulated in fibroblasts in the human fibrostenotic intestine. Our study also revealed that fibroblastic PTGIS inhibits YAP/TAZ via PGI2 in an autocrine manner, which suggests a loss of local autocrine PGI2-PTGIR signaling in intestinal fibrosis. Importantly, a previous DNA methylation analysis of Crohn’s disease-associated fibrosis revealed that DNA methylation of the *PTGIS* gene is a fibrosis-specific event.^59^ DNA methylation might drive fibrosis by silencing antifibrotic genes, and TGF-β can promote fibrosis by inducing global alterations in DNA methylation and epigenetic downregulation of antifibrotic genes.^60–62^ Thus, TGF-β-induced epigenetic inhibition could account for our finding that TGF-β suppresses *PTGIS* gene transcription in intestinal fibroblasts. In addition, BMP2 and BMP9 attenuate TGF-β-induced fibrosis in various organs.^43–46^ Here, we revealed that BMP2/9-SMAD5 signaling antagonizes TGF-β-SMAD2/3 signaling to activate *PTGIS* gene transcription in intestinal fibroblasts. Therefore, restoring or enhancing the expression of PTGIS in pathogenic fibroblasts via BMP2/9 treatment or DNMT inhibitors could be an alternative therapeutic strategy for reactivating PGI2-PTGIR signaling to treat intestinal fibrosis.

In healthy noninflamed mucosa from IBD patients, PGI2 levels are significantly lower than those in unhealthy inflamed mucosa from the same patients or control individuals.^30^ This finding is consistent with our observation that serum PGI2 levels in CD patients are lower than those in healthy controls. In addition, our findings suggest that the serum PGI2 level could be a predictor of fibrostenotic Crohn’s disease. More importantly, the serum PGI2 level decreases with prolonged disease duration, which might reflect the development of intestinal fibrosis and further support the rationale of restoring PGI2-PTGIR signaling to prevent and treat CD-associated strictures.

Notably, in contrast to the decreasing serum PGI2 levels, PGI2 levels were dramatically increased in CD patients with fistulas in our cohort. Previous studies reported differences in the migration of colonic lamina propria fibroblasts from CD patients compared to healthy people.^63^ Compared with that of CD-CLPF, the migration ability of fistula-CLPF was significantly reduced, whereas the migration potential of fibrosis-CLPF was increased.^63^ Given the strong promigratory function of YAP/TAZ in fibroblasts, elevated PGI2 levels in serum might account for the suppressed fibroblast migration caused by inactivation of YAP/TAZ, impairing wound healing and leading to subsequent fistula formation. It is worth noting that in CD, intestinal fibrosis is often markedly present even in fistulated intestinal tracts.^64^ The pathogenesis of surrounding fibrosis in CD fistulas might be different from that of intestinal fibrosis in intestinal strictures. Thus, it could be infeasible to assess the fibrostenotic development in CD patients with fistulas by using serum PGI2 levels.

The limitations of this study include the retrospective nature of the study and the small number of serum CD samples. A multicenter study should be conducted to determine the optimal threshold of PGI2 to predict CD-associated stricturing or penetrating complications. Another limitation of this study is that although we detected PGI2 levels in intestinal tissue specimens and observed a correlation between PTGIS expression and prostacyclin biosynthesis in intestinal strictures, the small sample size restricted the statistical power. Future studies are needed to validate these clinical associations in a large cohort.

Taken together, our findings demonstrate that PGI2 production is reduced and that PGI2-PTGIR signaling is lost during the development of intestinal fibrosis. The increased expression level of PTGIR caused by the major inflammatory cytokine TNF-α in pathogenic fibroblasts and the absence of PTGIR expression in intestinal epithelial cells suggest that PTGIR is a promising target for the specific inhibition of YAP/TAZ in intestinal fibroblasts. Hence, fibroblast-specific targeting of YAP/TAZ via PTGIR agonism represents a pharmacologically tractable approach to reverse intestinal fibrosis in CD (Graphical abstract).

## Supplementary Material

jjaf084_suppl_Supplementary_Tables_S1-S5_Figures_S1-S5

## Data Availability

There are no new data associated with this article.
