# Correction to: Restoring Prostacyclin/PGI2-PTGIR signaling alleviates intestinal fibrosis in Crohn’s disease via fibroblast-specific YAP/TAZ inhibition

**DOI:** 10.1093/ecco-jcc/jjaf142

**Published:** 2025-12-22

This is a correction to: Weijun Ou, Yaosheng Wang, Weimin Xu, Zhebin Hua, Xiaolei Wang, Wensong Ge, Wenjun Ding, Yingwei Chen, Chen-Ying Liu, Peng Du, Restoring Prostacyclin/PGI2-PTGIR signaling alleviates intestinal fibrosis in Crohn’s disease via fibroblast-specific YAP/TAZ inhibition, *Journal of Crohn's and Colitis*, Volume 19, Issue 6, June 2025, https://doi.org/10.1093/ecco-jcc/jjaf084

The following emendations of typographical error have been made to the originally-published manuscript.

In the Graphical Abstract, “TGF-α” is corrected to “TNF-α”.

In Figure 1A, “PG12” is corrected to “PGI2”.

In Figure 2C, “OTGF-β1” is corrected to “TGF-β1”.

“[team please remove these brackets and red text and replace with text and image in the attached file supplementary cx.docx file]”.


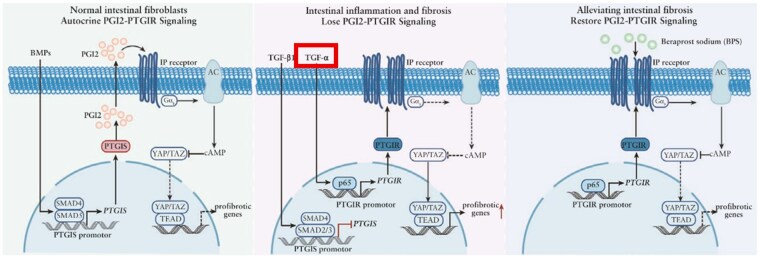


TGF-α should be corrected to **TNF-α** in Graphical Abstract.


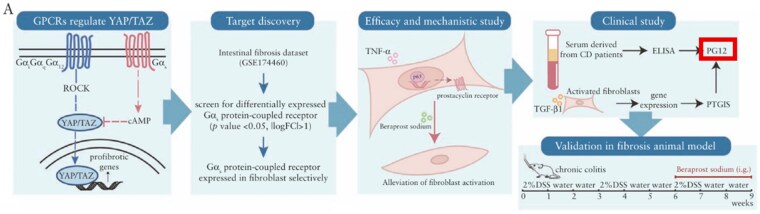


PG12 should be corrected to **PGI2** in Figure 1A.


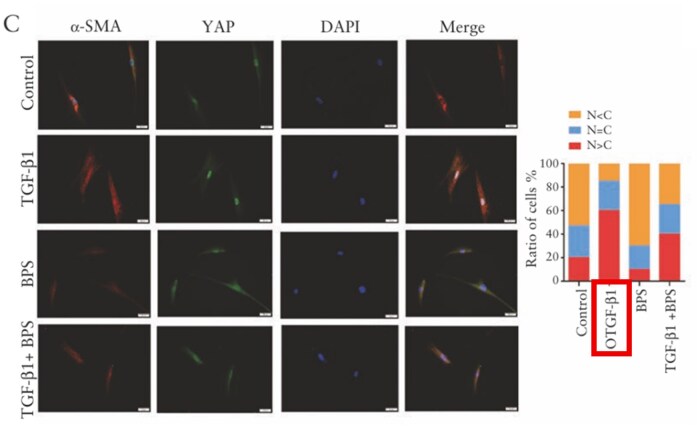


OTGF-β1 should be corrected to **TGF-β1** in Figure 2C.

In Figure S4B, the colorectal length from mice subjected to different treatments were originally measured from cecum to anus. Colorectal length should be measured from ileocecal region to anus and change of the measuring method doesn’t change the result and conclusion in Figure S4B. The correct barplot of colorectal length was updated in the Supplement files during the proof process. Consequently, the corresponding bar chart is updated here.

Figure S4B. Colorectal length from mice subjected to different treatments.

