# Supplementary material for: Restoring Prostacyclin/PGI2-PTGIR signaling alleviates intestinal fibrosis in Crohn’s disease via fibroblast-specific YAP/TAZ inhibition
Source: J Crohns Colitis. 2025 May 20;19(6):jjaf084. doi: 10.1093/ecco-jcc/jjaf084 (PMC12138779; doi:10.1093/ecco-jcc/jjaf084)
Supplement: jjaf084_suppl_Supplementary_Tables_S1-S5_Figures_S1-S5 [file jjaf084_suppl_supplementary_tables_s1-s5_figures_s1-s5.docx]

**Supplemental information titles and legends**

**Figure S1. Effect of the PTGIR agonist BPS on the localization of YAP in intestinal fibroblasts with PTGIR knockdown.** (A) Intestinal fibroblasts derived from CD patients were transfected with siNC or PTGIR siRNA for 24 hours and then treated with TGF-β1 (10 ng/ml, 24 hours) with or without the PTGIR agonist BPS. YAP localization was detected via immunofluorescence staining.

**Figure S2. Positive correlation between PGI2 levels and PTGIS expression levels in intestinal tissues.** (A) PGI2 levels in the 13 paired stenotic and nonstenotic intestines were compared. (B) PTGIS expression levels in the 13 paired stenotic and nonstenotic intestines were detected by western blot for semiquantitative analysis. (C) Pearson correlation analysis of the intestinal tissue PGI2 levels and PTGIS expression levels (n=26). Statistical analyses were performed via Student’s t test for (A). Significant differences are shown by ^***^p<0.001.

**Figure S3. PTGIS inhibits YAP/TAZ activation through PGI-PTGIR signaling and subsequent LATS inhibition.** (A) Intestinal fibroblasts derived from CD patients were transfected with siNC or PTGIS siRNA for 48 hours and then treated with PTGIR agonist BPS (10 µM, 24 hours) with or without LATS1/2 inhibitor VT02956 (2 µM, 2 hours). Indicated protein and phosphorylated-protein levels were detected by western blot.

**Figure S4. The PTGIR agonist BPS reverses intestinal inflammation in an acute colitis murine model.** (A) Schematic diagram of BPS treatment in a DSS-induced acute colitis model. The mice were treated with 3% DSS to induce acute inflammation and then with solvent or BPS via oral gavage from the third day to the seventh day. (B-D, F) Colorectal length (B), DAI score (C), representative images of hematoxylin‒eosin-stained sections (D) and Masson’s trichrome-stained sections (E) of the distal colon from mice subjected to different treatments are shown as indicated. Scale bar =50 μm (upper panels) and 20 μm (lower panels). (E, G) Representative immunohistochemical images of CD3 (E) and YAP (G) expression in colonic tissues from mice in different groups. A semiquantitative immunohistochemical score was used for statistical analysis. Scale bar =50 μm (upper panels) and 20 μm (lower panels). In all cases, the bars in the graphs represent the mean ± S.D. Statistical analyses were performed via Student’s t test for (C), the Mann‒Whitney test for histological scores and one-way ANOVA for (B, E, G). Significant differences are shown by ^*^p<0.05, ^**^p<0.01, and ^****^p<0.0001.

**Figure S5. Statistical analysis of the histological scores and semiquantitative immunohistochemical scores related to Figure 7.** (A, B) Inflammation scores (A) and fibrosis scores (B) of the distal colon in mice subjected to different treatments, as described in Figure 7. (C-F) Semiquantitative immunohistochemical scores of PTGIR, YAP, α-SMA and COL1A1 expression in the distal colon of mice subjected to different treatments were used for statistical analysis. In all cases, the bars in the graphs represent the mean ± S.D. Statistical analyses were performed via the Mann‒Whitney test for histological scores and one-way ANOVA for (C‒F). Significant differences are indicated by ^*^p<0.05, ^**^p<0.01 and ^***^p<0.001.

**Table S1.** Baseline characteristics of the CD patients included in this study.

**Table S2.** Analysis of the complications and clinical outcomes in CD patients stratified by PGI2 levels.

**Table S3.** Univariable and multivariate analyses of the risk factors for CD stenosis.

**Table S4.** The antibodies and reagents used in this study.

**Table S5.** The primers and related sequences used in this study.

**Supplemental information**

**
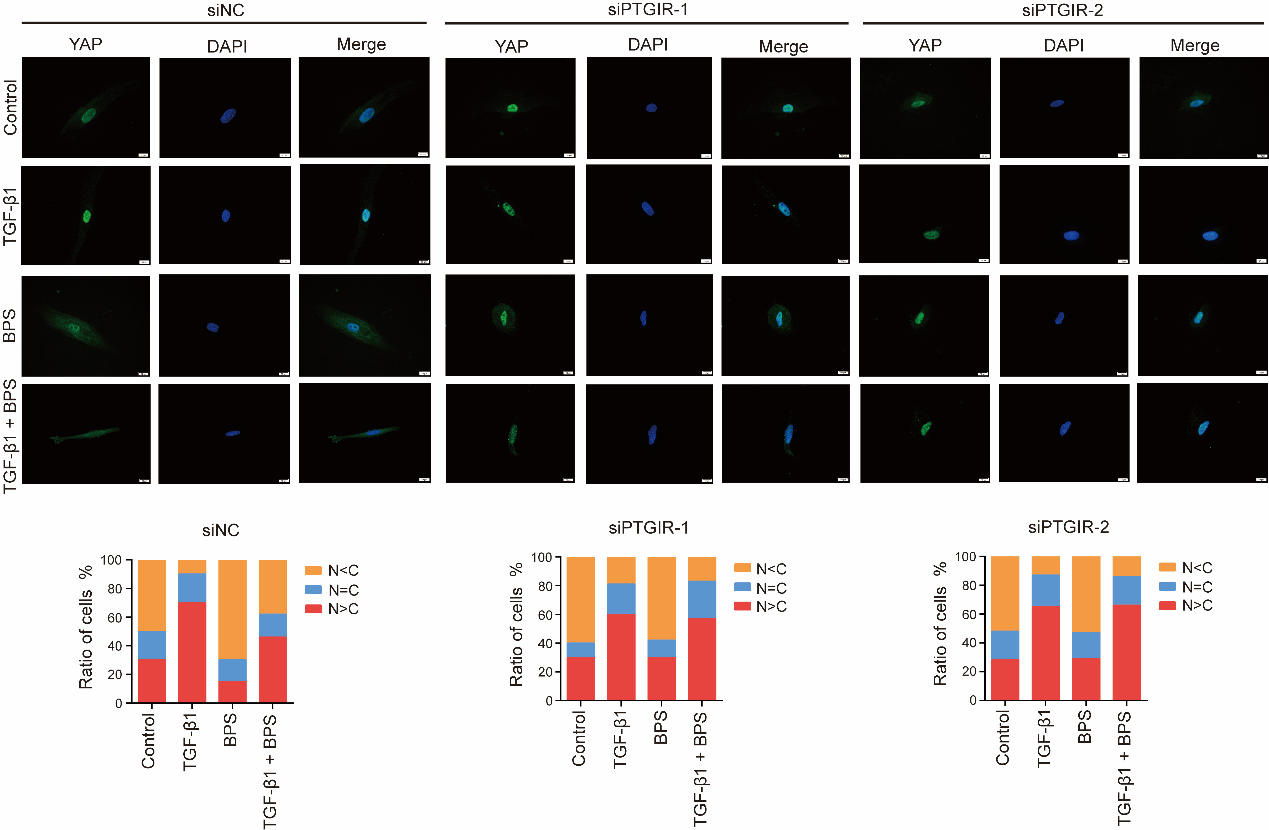
**

**Figure S1. Effect of the PTGIR agonist BPS on the localization of YAP in intestinal fibroblasts with *PTGIR* knockdown.** A. Intestinal fibroblasts derived from CD patients were transfected with siNC or *PTGIR* siRNA for 24 hours and then treated with TGF-β1 (10 ng/ml, 24 hours) with or without the PTGIR agonist BPS. YAP localization was detected via immunofluorescence staining.


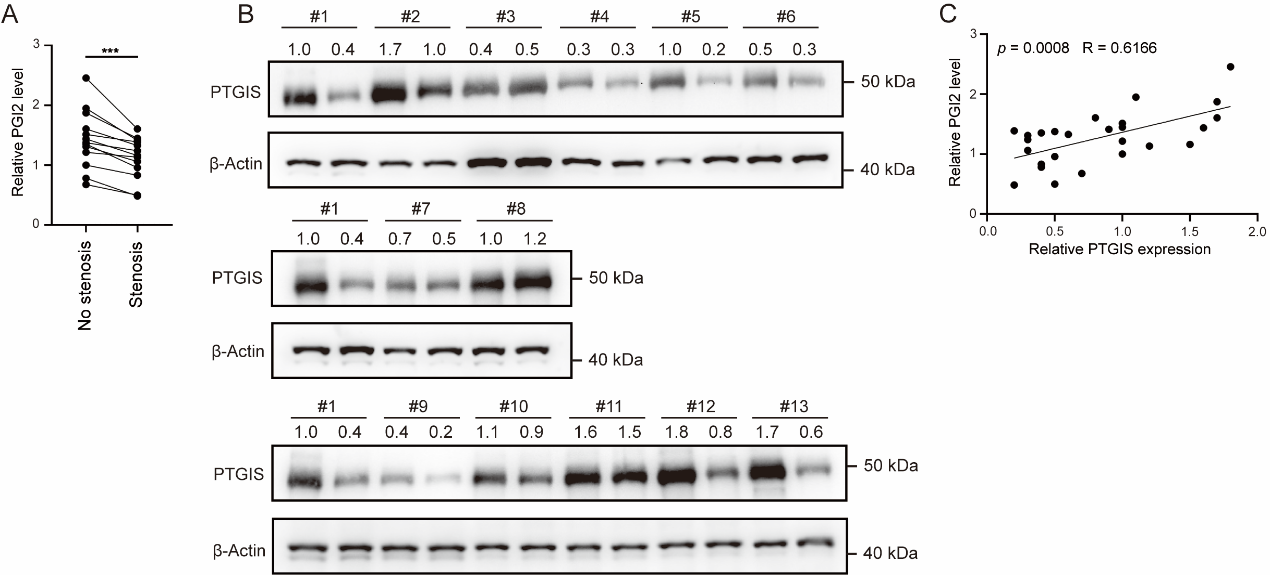


**Figure S2. Positive correlation between PGI2 levels and PTGIS expression levels in intestinal tissues.** (A) PGI2 levels in the 13 paired stenotic and nonstenotic intestines were compared. (B) PTGIS expression levels in the 13 paired stenotic and nonstenotic intestines were detected by western blot for semiquantitative analysis. (C) Pearson correlation analysis of the intestinal tissue PGI2 levels and PTGIS expression levels (n=26). Statistical analyses were performed via Student’s t test for (A). Significant differences are shown by ^***^p<0.001.


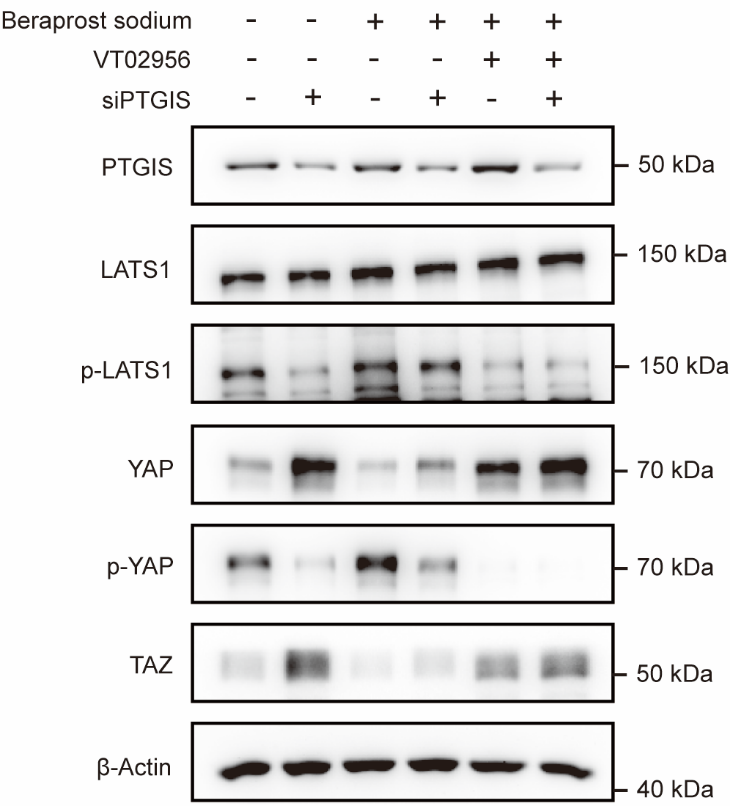


**Figure S3. PTGIS inhibits YAP/TAZ activation through PGI-PTGIR signaling and subsequent LATS inhibition.** (A) Intestinal fibroblasts derived from CD patients were transfected with siNC or PTGIS siRNA for 48 hours and then treated with PTGIR agonist BPS (10 µM, 24 hours) with or without LATS1/2 inhibitor VT02956 (2 µM, 2 hours). Indicated protein and phosphorylated-protein levels were detected by western blot.


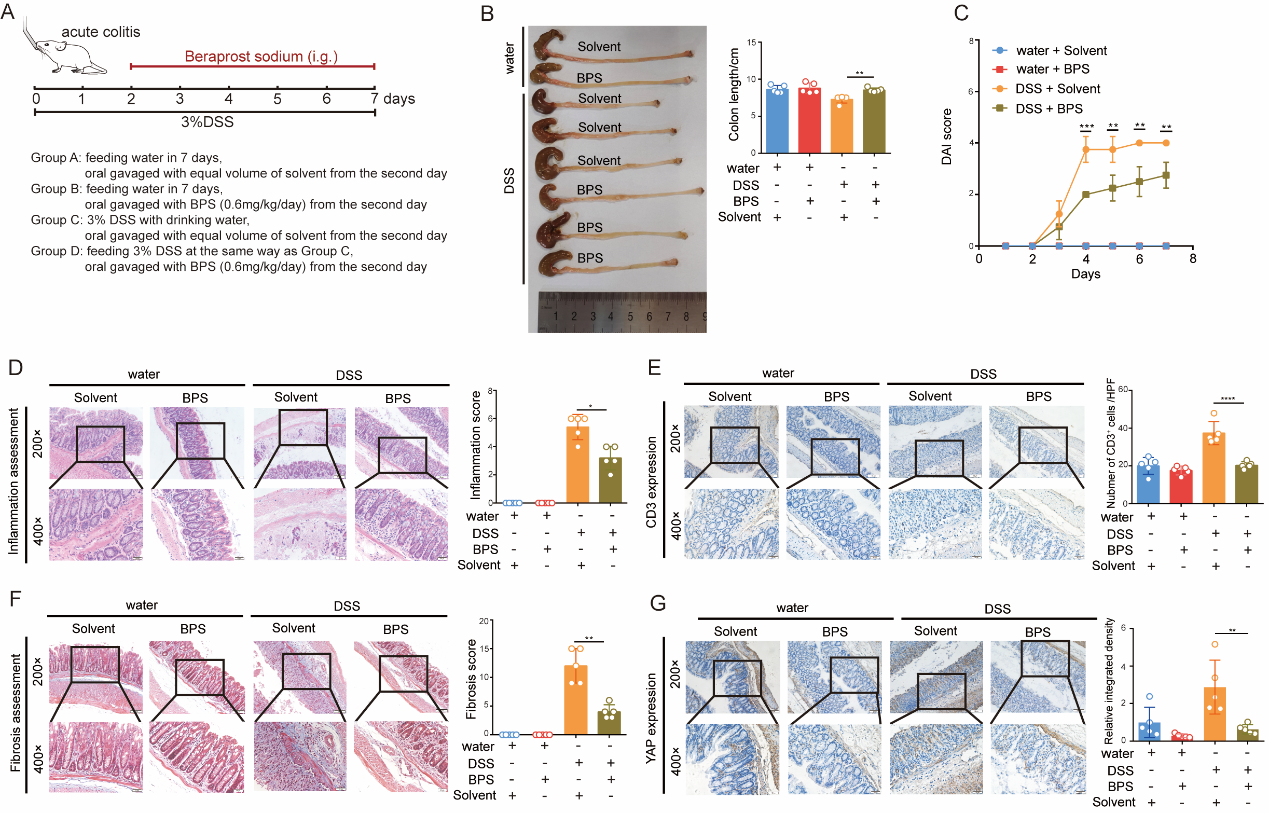


**Figure S4. The PTGIR agonist BPS reverses intestinal inflammation in an acute colitis murine model.** (A) Schematic diagram of BPS treatment in a DSS-induced acute colitis model. The mice were treated with 3% DSS to induce acute inflammation and then with solvent or BPS via oral gavage from the third day to the seventh day. (B-D, F) Colorectal length (B), DAI score (C), representative images of hematoxylin‒eosin-stained sections (D) and Masson’s trichrome-stained sections (E) of the distal colon from mice subjected to different treatments are shown as indicated. Scale bar =50 μm (upper panels) and 20 μm (lower panels). (E, G) Representative immunohistochemical images of CD3 (E) and YAP (G) expression in colonic tissues from mice in different groups. A semiquantitative immunohistochemical score was used for statistical analysis. Scale bar =50 μm (upper panels) and 20 μm (lower panels). In all cases, the bars in the graphs represent the mean ± S.D. Statistical analyses were performed via Student’s t test for (C), the Mann‒Whitney test for histological scores and one-way ANOVA for (B, F, G). Significant differences are shown by ^*^p<0.05, ^**^p<0.01, and ^****^p<0.0001.


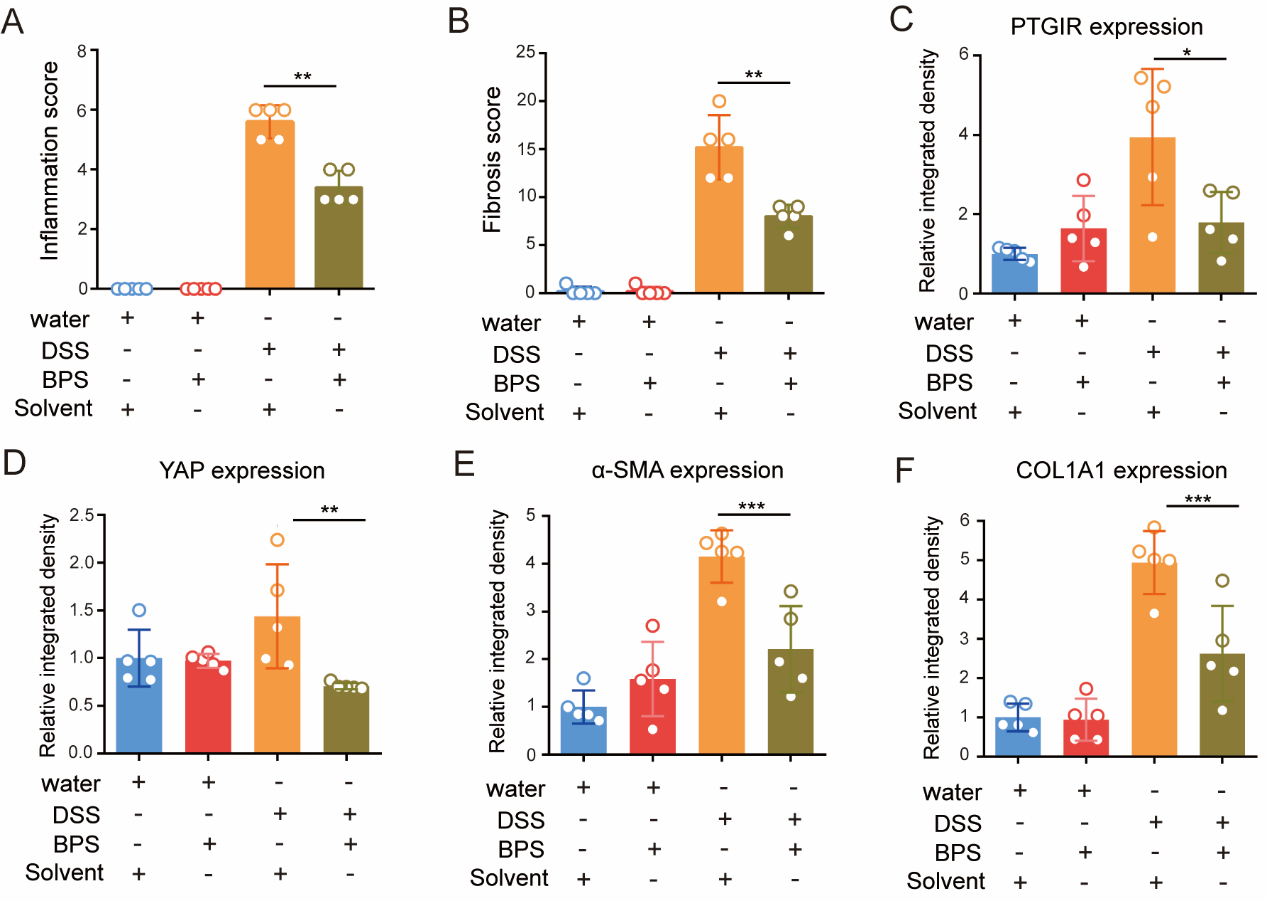


**Figure S5. Statistical analysis of the histological scores and semiquantitative immunohistochemical scores related to Figure 7.** (A, B) Inflammation scores (A) and fibrosis scores (B) of the distal colon in mice subjected to different treatments, as described in Figure 7. (C-F) Semiquantitative immunohistochemical scores of PTGIR, YAP, α-SMA and COL1A1 expression in the distal colon of mice subjected to different treatments were used for statistical analysis. In all cases, the bars in the graphs represent the mean ± S.D. Statistical analyses were performed via the Mann‒Whitney test for histological scores and one-way ANOVA for (C‒F). Significant differences are indicated by ^*^p<0.05, ^**^p<0.01 and ^***^p<0.001.

**Supplemental Tables**

**Table S1.** Baseline characteristics of the CD patients included in this study

| Characteristic | Total (n = 118) |
| --- | --- |
| Sex (male/female) | 65/53 |
| Age at diagnosis, n (%) |  |
| A1 | 13 (11.0) |
| A2 | 81 (68.6) |
| A3 | 24 (20.3) |
| Disease duration (yr, median (IQR)) | 3.0 (1.5-5.0) |
| Extraintestinal manifestations, n (%) | 62 (52.5) |
| Serious complication, n (%) | 71 (60.2) |
| Family history, n (%) |  |
| No | 118 (100) |
| Family history of CRC or IBD | 0 (0.0) |
| Family history of other autoimmune disease | 0 (0.0) |
| History of surgery, n (%) | 15 (12.7) |
| Indications for surgery, n (%) |  |
| Medical treatment failure | 3 (20.0) |
| Serious side effects of medication | 4 (26.7) |
| Intestinal obstruction | 5 (33.3) |
| Fistula | 0 (0.0) |
| Acute perforation | 2 (13.3) |
| Other acute serious complications | 1 (6.7) |
| Location of CD, n (%) |  |
| L1 | 24 (20.3) |
| L2 | 44 (37.3) |
| L3 | 49 (41.5) |
| L4 | 1 (0.8) |
| Behavior of CD, n (%) |  |
| B1 | 55 (46.6) |
| B2 | 49 (41.5) |
| B3 | 14 (11.9) |
| Mesalamine, n (%) | 61 (51.7) |
| Biologics, n (%) | 70 (59.3) |
| Steroids, n (%) | 40 (33.9) |
| Immunomodulators, n (%) | 25 (21.2) |
| SES-CD (score, median (IQR)) | 6.0 (4.0-12.0) |

*IQR* interquartile range, *CD* Crohn’s disease, *IBD* inflammatory bowel disease, *CRC* colorectal cancer, *SES-CD* Simple Endoscopic Score for Crohn’s disease

*A1*, *A2* and *A3* represent age at diagnosis subgroups according to the Montreal classification. *L1*, *L2*, *L3* and *L4* represent different locations of CD according to the Montreal classification. *B1*, *B2* and *B3* represent different behaviors of CD according to the Montreal classification.

**Table S2.** Analysis of the complications and clinical outcomes in CD patients stratified by PGI2 levels

| **Variables** | **PGI2 < 272.9** | **PGI2 ≥ 272.9** | ***p* value** |
| --- | --- | --- | --- |
|  | **n = 61** | **n = 57** |  |
| Intestinal stenosis, n (%) |  |  | < 0.001^a^ |
| No | 25 (41.0) | 44 (77.2) |  |
| Yes | 36 (59.0) | 13 (22.8) |  |
| Abscess, n (%) |  |  | 1.000 ^b^ |
| No | 58 (95.1) | 55 (96.5) |  |
| Yes | 3 (4.9) | 2 (3.5) |  |
| Fistula, n (%) |  |  | 0.002 ^b^ |
| No | 60 (98.4) | 45 (80.7) |  |
| Yes | 1 (1.6) | 11 (19.3) |  |
| EIMs, n (%) |  |  | 0.698 ^a^ |
| No | 30 (49.2) | 26 (45.6) |  |
| Yes | 31 (50.8) | 31 (54.4) |  |
| Surgery, n (%) |  |  | 0.892 ^a^ |
| No | 53 (86.9) | 50 (87.7) |  |
| Yes | 8 (13.1) | 7 (12.3) |  |

*EIMs* Extraintestinal manifestations.

^a^ Chi-square test

^b^ Fisher’s exact test

**Table S3.** Univariable and multivariate analyses of the risk factors for CD stenosis

| **Variables** | **Nonstenosis group**  **(n = 69)** | **Stenosis group**  **(n = 49)** | **Univariate**  ***p* value** | **Multivariate** | | |
| --- | --- | --- | --- | --- | --- | --- |
|  |  |  |  | **Odds Ratio** | **95% CI** | ***p* value** |
| Sex, n (%) |  |  | 0.710 ^a^ |  |  |  |
| Male | 39 (56.5) | 26 (53.1) |  |  |  |  |
| Female | 30 (43.5) | 23 (46.9) |  |  |  |  |
| Age at diagnosis, n (%) |  |  | 0.031 ^a^ | 0.450 | (0.149-1.357) | 0.156 ^a^ |
| ≤ 40 y | 49 (71.0) | 43 (87.8) |  |  |  |  |
| > 40 y | 20 (29.0) | 6 (12.2) |  |  |  |  |
| Disease duration, n (%) |  |  | 0.043 ^a^ | 0.964 | (0.376-2.473) | 0.939 ^a^ |
| < 3 y | 34 (49.3) | 15 (30.6) |  |  |  |  |
| ≥ 3 y | 35 (50.7) | 34 (69.4) |  |  |  |  |
| PGI2 secretion, n (%) |  |  | < 0.001 ^a^ | 4.772 | (1.981-11.494) | < 0.001 ^a^ |
| < 272.9 pg/ml | 25 (36.2) | 36 (73.5) |  |  |  |  |
| ≥ 272.9 pg/ml | 44 (63.8) | 13 (26.5) |  |  |  |  |
| Mesalamine, n (%) |  |  | 0.802 ^a^ |  |  |  |
| No | 34 (49.3) | 23 (46.9) |  |  |  |  |
| Yes | 35 (50.7) | 26 (53.1) |  |  |  |  |
| Steroids, n (%) |  |  | 0.878 ^a^ |  |  |  |
| No | 46 (66.7) | 32 (65.3) |  |  |  |  |
| Yes | 23 (33.3) | 17 (34.7) |  |  |  |  |
| Immunomodulators, n (%) |  |  | 0.862 ^a^ |  |  |  |
| No | 54 (78.3) | 39 (79.6) |  |  |  |  |
| Yes | 15 (21.7) | 10 (20.4) |  |  |  |  |
| Biologics, n (%) |  |  | 0.024 ^a^ | 1.994 | (0.794-5.008) | 0.142 ^a^ |
| No | 34 (49.3) | 14 (28.6) |  |  |  |  |
| Yes | 35 (50.7) | 35 (71.4) |  |  |  |  |

*PGI2* prostacyclin, CI confidence interval

^a^ Chi-square test

**Table S4.** The antibodies and reagents used in this study.

|  | | | |
| --- | --- | --- | --- |
| **Antibodies or reagents** | **Source** | **Identifier** | **RRID** |
| Anti-YAP | Santa Cruz Biotechnology | sc-101199 | AB_1131430 |
| Anti-YAP | Cell Signaling Technology | #14074 | AB_2650491 |
| Anti-YAP/TAZ | Cell Signaling Technology | #8418 | AB_10950494 |
| Anti-p-YAP (Ser127) | Cell Signaling Technology | #13008 | AB_2650553 |
| Anti-PTGIR | Abcam | ab196653 | - |
| Anti-PTGIS | Abcam | ab23668 | AB_2300427 |
| Anti-α-Smooth muscle actin | Abcam | ab5694 | AB_2223021 |
| Anti-β-actin | Cell Signaling Technology | #4967 | AB_330288 |
| Anti-Collagen Ⅰ（COL1A1） | Cell Signaling Technology | #72026 | - |
| Anti-CD3ε | Cell Signaling Technology | #78588 | - |
| Anti-p65 | Cell Signaling Technology | #8242 | AB_10859369 |
| Anti-SMAD2 | Cell Signaling Technology | #5339 | AB_10626777 |
| Anti-SMAD5 | Cell Signaling Technology | #12534 | AB_2797946 |
| DSS | MP Biomedicals | 160110 | - |
| TNF-α | R&D systems | 210-TA-100 | - |
| TGF-β1 | Peprotech | 100-21C-10 | - |
| BMP2 | MedChemExpress | HY-P7006 | - |
| BMP9 | MedChemExpress | HY-P700033AF | - |
| BPS | MedChemExpress | HY-13569A | - |

**Table S5.** The primers and related sequences used in this study.

|  | | | |
| --- | --- | --- | --- |
| **Target** | **Species** | **Forward Primer** | **Reverse Primer** |
| PTGIR | Human | ATGACAGTGGTCATGGCCGTGT | GTTGAAGGCGTAGAAGCGGAAG |
| PTGIS | Human | CCGTGGCTCCCTGTCAGT | GCAGCTTCCACAGGCGAC |
| CTGF | Human | CCTGCAGGCTAGAGAAGCAG | TGGAGATTTTGGGAGTACGG |
| CYR61 | Human | AAGAAACCCGGATTTGTGAG | GCTGCATTTCTTGCCCTTT |
| TNF-α | Human | CCTCTCTCTAATCAGCCCTCTG | GAGGACCTGGGAGTAGATGAG |
| PTGIR PEAK1 for CUT&RUN | Human | CCCTGACAACTGGACACCAA | GGACATTGCCCAGGGATTGA |
| PTGIR PEAK2 for CUT&RUN | Human | CAATCCCTGGGCAATGTCCT | ACTCTCCTCCCCACATCCTC |
| PTGIS for CUT&RUN | Human | GATGGGGAAACAGCCAAGGA | AGGTGAGGATGAGGGGACTC |
| ACTA2 | Human | AAGAGGAATCCTGACCCTGAA | TGGTGATGATGCCATGTTCT |
| GAPDH | Human | GGGTGATGCAGGTGCTACTT | GGCAGGTTTCTCAAGACGGA |
| **siRNA sequences** | | |  |
| **Name** | **Sequence (5' to 3')** | |  |
| siPTGIR1 | CCCATCCATCTCATTGTCTAA dTdT | |  |
| siPTGIR2 | CTTCCGCTTCTACGCCTTCAA dTdT | |  |
| siPTGIS1 | GCTGATGTCTTCCACACCTTT dTdT | |  |
| SiPTGIS2 | CCCAGAGGTATTTAAATACAA dTdT | |  |
